# CXCL12/CXCR4 axis governs Treg spatial dominance over CD8+ T cells via IL-2 sequestration: a dual therapeutic target in prostate cancer

**DOI:** 10.3389/fimmu.2025.1626708

**Published:** 2025-07-08

**Authors:** Junyi Li, Long Zhang, Ruoyang Liu, Changwen Xu, HuiHui Tang, Yunfei Zou, Qingfei Cao, Weichao Huang

**Affiliations:** ^1^ Department of Urology, The First Affiliated Hospital of Jinzhou Medical University, Jinzhou, Liaoning, China; ^2^ Department of Urology, The First Affiliated Hospital of Zhengzhou University, Zhenzhou, Henan, China; ^3^ Department of Cardiology, The First Affiliated Hospital of Jinzhou Medical University, Jinzhou, Liaoning, China

**Keywords:** prostate cancer, tumor immune microenvironment, CD8+ T cell, regulatory T cells, CXCL12/CXCR4 axis, IL-2/STAT5 signaling

## Abstract

**Background:**

Prostate cancer (PCa) is characterized by high incidence and recurrence rates, presenting as an immune ‘cold’ tumor that exhibits a poor response to immunotherapy. The mechanisms underlying immune suppression and evasion within the tumor microenvironment (TME) of PCa remain inadequately understood.

**Methods:**

A comprehensive analysis of the immune environment in PCa was conducted using combined single-cell and spatial transcriptomic approaches, encompassing samples from healthy tissue, adjacent normal tissue, and localized tumors. Cell abundance and polarization state analyses were performed to identify pivotal cellular populations. Spatial deconvolution techniques were employed to elucidate cell composition within its spatial context. Additionally, cell niche and spatial colocalization analyses were conducted to evaluate potential cellular interactions. Immune response enrichment analysis was utilized to assess cellular response states. *In vivo* and *in vitro* experiments were conducted to validate hypotheses.

**Results:**

Data indicated a prevalent immunosuppressive state among CD8 T cells, accompanied by variations in cell abundance. Macrophages emerged as key regulators in recruiting CD8+ effector T cells and regulatory T cells (Tregs) into the TME, mediated by the CXCL12/CXCR4 axis. A spatial proximity relationship was established between CD8+ effector T cells and Tregs, suggesting Tregs directly influence CD8+ T cell function. Immune cell state analysis revealed interleukin-2 (IL-2) as a critical cytokine in reshaping the immune microenvironment, with Tregs competitively depleting IL-2 and mediating IL-2/STAT5 signaling to induce CD8+ effector T cell exhaustion. Treatment with CXCR4 inhibitor and IL-2 demonstrated significant antitumor effects and reversed immune dysfunction in both *in vivo* and *in vitro* experiments, with combined treatment exhibiting superior efficacy.

**Conclusion:**

These findings elucidate the role of macrophages in mediating the CXCL12/CXCR4 axis to aggregate CD8+ effector T cells and Tregs, thereby influencing the TME. Furthermore, Tregs competitively deplete IL-2 and mediate IL-2/STAT5 signaling, leading to CD8+ effector T cells exhaustion and the establishment of an immunosuppressive microenvironment.

## 1 Introduction

Prostate cancer (PCa) is one of the most prevalent malignancies among men globally, ranked as the second most common type and the fifth leading cause of cancer-related mortality in males (1). Despite advances in treatment modalities, including androgen deprivation therapy (ADT), targeted therapies, and chemotherapy, the impact on overall cure rates remains limited, alongside a notably high rate of biochemical recurrence (2). Although ADT effectively controls early-stage hormone-sensitive prostate cancer (HSPC), approximately 25%-30% of patients progress to castration-resistant prostate cancer (CRPC) within five years, further evolving into metastatic CRPC (mCRPC) (3, 4). Targeted therapies have shown some promise in prolonging overall survival (OS) and progression-free survival (PFS); however, effective responses are observed in only a subset of PCa patients (5). Consequently, immunotherapy has emerged as a promising approach in cancer management, aiming to identify common response characteristics within the tumor microenvironment (TME).

PCa is often characterized as an immune ‘cold’ tumor, exhibiting immunosuppressive properties due to a paucity of immune cells within the TME, which contributes to its poor response to immunotherapeutic strategies (6). Consequently, checkpoint blockade therapies, such as anti-PD-1/PD-L1 and anti-CTLA-4 agents, are currently not preferred treatments in this context, as they demonstrate limited efficacy. The complex immune cellular landscape within the TME of PCa remains poorly understood, necessitating deeper exploration of the cellular interactions and variations in immune populations that play pivotal roles in determining the effectiveness of immunotherapies (7). Thus, there is an urgent need to elucidate the immune cell states and interactions within the TME, with the goal of transitioning the immune response from ‘cold’ to ‘hot’ (8). Identifying a common immunosuppressive mechanism to reshape the TME could enhance the efficacy of immunotherapy and expand the benefits to more PCa patients.

Recent advancements in high-throughput sequencing technologies, including single-cell and spatial transcriptomics, have been rapidly integrated into PCa research to elucidate underlying cellular mechanisms within the TME at high resolution (8, 9). In this study, single-cell RNA sequencing data obtained from improved tissue dissociation and library preparation techniques were utilized to retain and preserve a diverse array of immune cells, providing a comprehensive depiction of the TME landscape in PCa. Additionally, paired spatial transcriptome data from Slide-seq V2 was employed to contextualize the spatial distribution of TME populations, particularly focusing on the proximity of key immune cells.

Through the integrated analysis of single-cell and spatial transcriptomic data, this study aims to reveal the characteristics of the immune microenvironment and enhance our understanding of the modulatory mechanisms underlying the immunosuppressive TME in PCa. Our observations indicate (1): CD8 T cell subpopulations frequently exhibit signs of exhaustion (2); there is an elevation in macrophage proportions and their interaction with lymphocytes within tumor regions (3); macrophages mediate the CXCL12/CXCR4 axis, facilitating the recruitment of regulatory T cells (Tregs) and CD8+ effector T cells to exert anti-tumor effects (4); spatial colocalization between Tregs and CD8+ effector T cells has been identified (5); the activation state of immune cells is confirmed, with IL-2 cytokines playing a crucial role in shifting the immune microenvironment from ‘cold’ to ‘hot’ (6); Tregs compete for IL-2, inducing CD8+ T cell exhaustion and promoting tumor progression. This careful and insightful dissection offers novel perspectives on the molecular mechanisms and cellular crosstalk within the TME, potentially guiding the development of new therapeutic targets and improving the efficacy of immunotherapy for PCa patients.

## 2 Materials and methods

### 2.1 Comprehensive single-cell data processing

After excluding samples with low sequencing saturation, a total of 36 scRNA-seq datasets, encompassing 149,200 cells, were analyzed. These datasets included 4 from healthy prostate tissues, 14 from adjacent normal tissues paired with localized prostate cancer (PCa) samples, and 18 from localized PCa tissues. Enhanced library construction strategies were employed to capture a greater diversity of immune cell populations within the tissue, including healthy, adjacent normal, and tumor single-cell RNA sequencing (scRNA-seq) data (10). The ‘Seurat’ R package (4.3.2 version) was used to conduct downstream analysis. Rigorous quality control measures were implemented: cells expressing < 400 genes or yielding raw counts < 800 were excluded from further analysis. The R packages ‘doubletFinder’ and ‘decontX’ were utilized to identify potential doublet cells and contaminants from environmental RNA (11, 12). Subsequently, the top 2,000 highly variable genes were selected for principal component analysis (PCA). To mitigate batch effects, the ‘harmony’ algorithm was employed, and cell clusters were constructed at a resolution of 0.6, based on the K-nearest neighbors (KNN) graph. Cell annotation was performed according to marker genes identified in the original research (10).

### 2.2 Tumor cell identification

To accurately identify tumor cells and their epithelial subtypes, all epithelial cells were processed using the same methodology described previously. Different epithelial subtypes were annotated based on classical marker genes and associated enriched pathways. The ‘infercnvpy’ software, a Python implementation of the ‘infercnv’ R package, was employed to detect malignant cells within the epithelial population. Healthy epithelial cells served as reference cells, with other parameters set to default values. Based on copy number variation (CNV) scores, the Leiden algorithm was utilized to cluster the tumor cells. Additionally, the ‘monocle2’ and ‘PAGA’ packages were used to investigate the differentiation trajectory of the cell populations.

### 2.3 Analysis of key immune cell abundance and state

To further ascertain the abundance and state of lymphocytes, the observed/expected (O/E) ratio was employed to explore the tissue preferences of each lymphocyte subtype population. To assess the distribution preference of specific cell subtypes across tissues or conditions, we utilized the observed-to-expected (O/E) ratio analysis. The expected cell count for each subtype in a given tissue was calculated under the null hypothesis of random distribution using the formula: Eij = (ri × cj)/N, where ri and cj denote the total cell counts for subtype i and tissue j, respectively, and N is the total cell count across all tissues and subtypes. The O/E ratio (Ro/eij) was defined as: Ro/eij = Oij/Eij, with values greater than 1 indicating enrichment and less than 1 indicating depletion. Statistical significance was evaluated using chi-square or Fisher’s exact tests to validate deviations from the expected distribution (13, 14). This approach provides a quantitative framework for interpreting cell subtype-specific dynamics within tissues or experimental conditions. Subsequently, the ‘miloR’ package was utilized to calculate differential abundance based on a KNN graph, comparing healthy, adjacent normal, and tumor groups (15). Additionally, the ‘augur’ package was applied to elucidate cell type prioritization; this tool employs a machine learning model to predict cell states based on gene expression profiles (16). Parameters were set in accordance with the official tutorial guidelines. To evaluate cell states, gene sets associated with T cell cytotoxicity, exhaustion, regulatory T cells (Tregs), and M1/M2 macrophage polarization were curated and quantified using the ‘AddModuleScore’ function. The complete list of genes is provided in **Supplementary Table 1**.

### 2.4 Tumor microenvironment composition and prognostic analysis in the bulk RNA-seq cohort

To elucidate the TME composition from the Bulk RNA-seq data, we employed a deconvolution algorithm grounded in our Prostate Cancer Single Cell Atlas. The ‘BayesPrism’ R package was utilized, which models a prior distribution based on cell type-specific expression profiles derived from scRNA-seq (17). This approach allows for the joint estimation of the posterior distribution of cell type composition and cell type-specific gene expression from bulk RNA-seq data of tumors in the TCGA-PRAD cohort. Genes with low expression, including housekeeping genes, were filtered out to enhance the analysis. We specifically selected protein-coding genes and highly variable genes to optimize calculation efficiency and accuracy. The estimated cell abundances of candidate cell types were then employed in survival analyses within the TCGA-PRAD cohort to assess their prognostic significance. Specifically, the ‘survminer’ R package was utilized to objectively calculate the optimal cutoff values for stratifying patients into high- and low-abundance groups. This approach minimizes subjective bias by employing statistical algorithms to identify thresholds that most effectively distinguish between groups in relation to survival outcomes.

### 2.5 Cell niche analysis in the spatial transcriptome

We used Slide-seq V2 spatial transcriptome datasets from original research, comprising four healthy prostate samples, five matched adjacent normal tissues, and four localized prostate cancer lesions. Beads with fewer than 500 unique molecular identifiers (UMIs) or fewer than 200 detected genes were excluded during quality control. The remaining spots were normalized and carried forward into all downstream analyses. To investigate cell composition and niches at the spatial level, we utilized the ‘cell2location’ Python software to deconvolute scRNA-seq data within the Slide-seq spatial dataset, which comprised paired samples from different tissue sources. The analysis was conducted using raw count data, excluding genes with low expression. The parameter settings adhered to the slide-seq V2 analysis pipeline established in the original research on algorithm development (18). The Q05 posterior distribution weight matrix calculated by ‘cell2location’ represents the inferred cell-type abundances across spatially resolved locations. This matrix encapsulates probabilistic cell distribution within the spatial context, highlighting the relative contribution of various cell types at each spatial coordinate. To further analyze these spatial-level data, a KNN algorithm is used to calculate the cell type composition based on the Q05 posterior distribution. The KNN algorithm allows for the aggregation of cell type information, accounting for local neighborhood relationships within the spatial framework. By leveraging this method, one can estimate the composition of cell types at specific spatial locations by incorporating spatial proximity and context, thereby gaining deeper insights into tissue organization and cellular heterogeneity. The nearest 20 beads were regarded as a cell community, after which the Louvain algorithm was employed to cluster the cell niches within the Slide-seq data. Additionally, the Wilcoxon test was applied to assess variations in cell abundance between different niches, with a false discovery rate (FDR) threshold < 0.05 considered indicative of significant differences.

### 2.6 Spatial colocalization analysis

To identify the spatial relationships among different cell types, we utilized the ‘mistyR’ R package, which is based on an explainable machine learning framework designed to analyze highly multiplexed spatial data (19). All cell compositions from the various tissue groups were incorporated into the calculation framework as predictors and targets separately. The analysis highlighted the importance of spatial relationships among the cells. Notably, the variations observed between healthy and tumor samples may reveal potential mechanisms underlying tumorigenesis or progression at the spatial level.

### 2.7 Cell communication analysis

To reveal cellular interactions among cell types in the TME, we applied the ‘CellChat’ R package to infer potential cell communication between different cell clusters (20). Significant ligand-receptor (L-R) pairs were calculated based on gene expression profiles using the CellChat human database at the single-cell level. Potential tumorigenesis-related L-R pairs were identified by comparing the number and strength of interactions between healthy and tumor groups.

### 2.8 Immune response enrichment analysis

To decipher the cell response states of effector, tissue-resident CD8+ T cells, and Tregs, we utilized an immune dictionary derived from scRNA-seq data of mouse lymph nodes, which includes various immune cell response states across 86 cytokines. To accurately calculate differentially expressed genes (DEGs) of target cell types between tumor and healthy tissue groups, pseudobulk that is a superior method to balance the technical noise and biological insight for candidate cells were constructed based on different samples (21). The ‘edgeR’ package was employed to conduct differential expression analysis (DEA) using the raw count data. DEGs with p-values < 0.05 were used as the input matrix to calculate cosine similarity with the gene expression matrix from normalized scRNA-seq data related to different cytokine responses of target cells. The FDR method was applied to adjust the p-values, ensuring the accuracy of the calculation results. All steps adhered to the methodology outlined in the original study on immune response enrichment analysis (22).

### 2.9 Generation and treatment of PCa engraftment tumors in mice

C57BL/6 male mice, aged six weeks, were utilized for this study following approval from the Experimental Animal Ethics Committee of Jinzhou Medical University and Zhengzhou University. The mice were maintained under controlled conditions at 22°C with an average humidity of 55%, on a 12-hour light/12-hour dark cycle, with unrestricted access to food and water. Following anesthesia, prostate tissue was exposed, and RM1 cells (1 × 10^6^) were injected into the left, right, and dorsal lobe membranes of the prostate using microsyringes. The incisions were subsequently sutured. After a seven-day stabilization period, the mice received intraperitoneal treatments with phosphate-buffered saline (PBS) and AMD3100 octahydrochloride (ab120718). AMD3100 (Plerixafor, ab120718) was administered subcutaneously at a dose of 10 mg/kg once daily for one week, with treatments delivered at designated time points (23). Tumor size was assessed using an *in vivo* imaging system, and tumor tissue was collected for flow cytometry analysis.

### 2.10 Immune lymphocyte flow analysis

Tumor tissue was harvested and homogenized, then re-suspended in PBS. Lymphocytes were isolated from the tumor using a lymphocyte isolation solution. Following the determination of cell concentration, the following antibodies were added to characterize Tregs: APC-conjugated anti-mouse CD4 antibody (Biolegend^®^, 100411), PerCP-conjugated anti-mouse CD25 antibody (Biolegend^®^, 102027), and FOXP3 monoclonal antibody (Thermo Fisher, Catalog # 11-5773-82). Additionally, FITC-conjugated anti-mouse CD3 antibody (Biolegend^®^, 100203) and PerCP/Cyanine5.5-conjugated anti-mouse CD8b.2 antibody (Biolegend^®^, 140417) were utilized to identify CD8+ T lymphocytes.

### 2.11 ELISA-based quantification of cytokines

The concentrations of cytokines IL-2, IL-4, IL-7, IL-10, and IL-15 were quantified using enzyme-linked immunosorbent assay (ELISA) kits specific to human cytokines, purchased from MEIMIAN, China. All experiments were conducted following the manufacturer’s protocol with minor optimizations to ensure precision and reproducibility. Samples and reference standards were carefully dispensed into the wells of an ELISA plate, followed by gentle mixing to ensure homogeneity. The plate was then sealed with adhesive film and incubated at 37°C for 30 minutes to facilitate antigen binding. A concentrated washing buffer was prepared by diluting it 30-fold with distilled water and used throughout the wash steps. After incubation, the adhesive film was removed, and the wells were emptied before being gently blotted dry. Each well underwent five cycles of washing, consisting of adding the washing buffer, standing for 30 seconds, and subsequent drying to remove unbound material. Next, 50 μL of enzyme-conjugated reagent was added to each well, except the blank control, followed by resealing the plate and incubating it at 37°C for another 30 minutes. After the incubation, the same washing procedure was repeated to eliminate excess enzyme-conjugated reagent. For color development, 50 μL of color reagent A and 50 μL of color reagent B were added to each well, gently mixed, and incubated in the dark at 37°C for 10 minutes. The reaction was subsequently stopped by the addition of 50 μL of stop solution, which caused the color of the solution to change from blue to yellow. The optical density (OD) of each well was measured at 450 nm using an ELISA plate reader within 15 minutes of adding the stop solution to ensure accuracy. Cytokine concentrations were calculated based on standard curves generated from known concentrations of reference standards. These values were expressed in picograms per milliliter (pg/mL), allowing quantitative comparisons between experimental groups.

### 2.12 Co-culture and apoptosis detection

Fresh mouse spleen lymphocytes were isolated using the Mouse Spleen Lymphocyte Isolation Kit (P8860, Solarbio), following the manufacturer’s instructions, and assessed for viability and activity to ensure optimal cellular health. Prostate tumor cells (RM1) were cultured to the logarithmic growth phase, digested into a single-cell suspension, and seeded in the bottom compartment of a Transwell plate (Corning^®^, 3462). Spleen lymphocytes were seeded into the upper chamber at an effector-to-target ratio (E:T) of 10:1, determined via preliminary optimization. The Transwell insert (0.4 μm pore size) facilitated indirect co-culture, allowing cytokines and soluble factors to mediate immune-tumor interactions. Cells were cultured in DMEM supplemented with 10% FBS, with recombinant IL-2 (10 ng/mL) added to activate immune cells as required, under standard conditions (37°C, 5% CO_2_). After 24 hours of co-culture, tumor cells were harvested and analyzed for apoptosis using the BD Pharmingen™ FITC Annexin V Apoptosis Detection Kit I, following the manufacturer’s instructions, and quantified via flow cytometry. Appropriate controls, including tumor and immune cells cultured separately, were included to ensure experimental robustness and reliability.

### 2.13 Generation and treatment of PCa subcutaneous model in mice

Six-week-old C57BL/6 male mice were utilized for this study. The mice were housed at 22°C with an average humidity of 55%, under a 12-hour light/12-hour dark cycle, and had unrestricted access to food and water. In accordance with ethical guidelines, the maximum size of the subcutaneous tumor was limited to 2 cm. RM1 cells (1 × 106) were subcutaneously injected into the mice, mixed with a Matrigel solution (1:1 ratio of PBS to Matrigel, BD Biosciences). Once the subcutaneous tumor reached approximately 100 mm³, mice were treated with IL-2 (MCE, HY-P7077) via intraperitoneal injection and/or AMD3100 octahydrochloride. AMD3100 was administered subcutaneously at a dose of 10 mg/kg once daily for one week, with all the treatments carried out at the specified time points. At the conclusion of the treatment, the mice were euthanized, and tumor grafts were excised for further analysis.

### 2.14 Statistical analysis

The processing and statistical analysis of transcriptome data were performed using R software (Version 4.3.2). Cell abundance among T cell subclusters was analyzed with the Wilcoxon test, considering a FDR < 0.05 as indicative of significant differences in the KNN graph. Log-rank tests and Kaplan–Meier survival analyses were employed to assess the prognostic impact of various CD8+ T cell subtypes within the TCGA-PRAD cohort. Experimental data were processed using GraphPad Prism (Version 9.4.1), and group comparisons were conducted using appropriate statistical methods, including t-tests, one-way analysis of variance. All experiments were conducted in triplicate or more. A P value < 0.05 was deemed statistically significant in this study.

## 3 Results

### 3.1 Landscape of the prostate cancer tumor microenvironment

**Figure 1:**
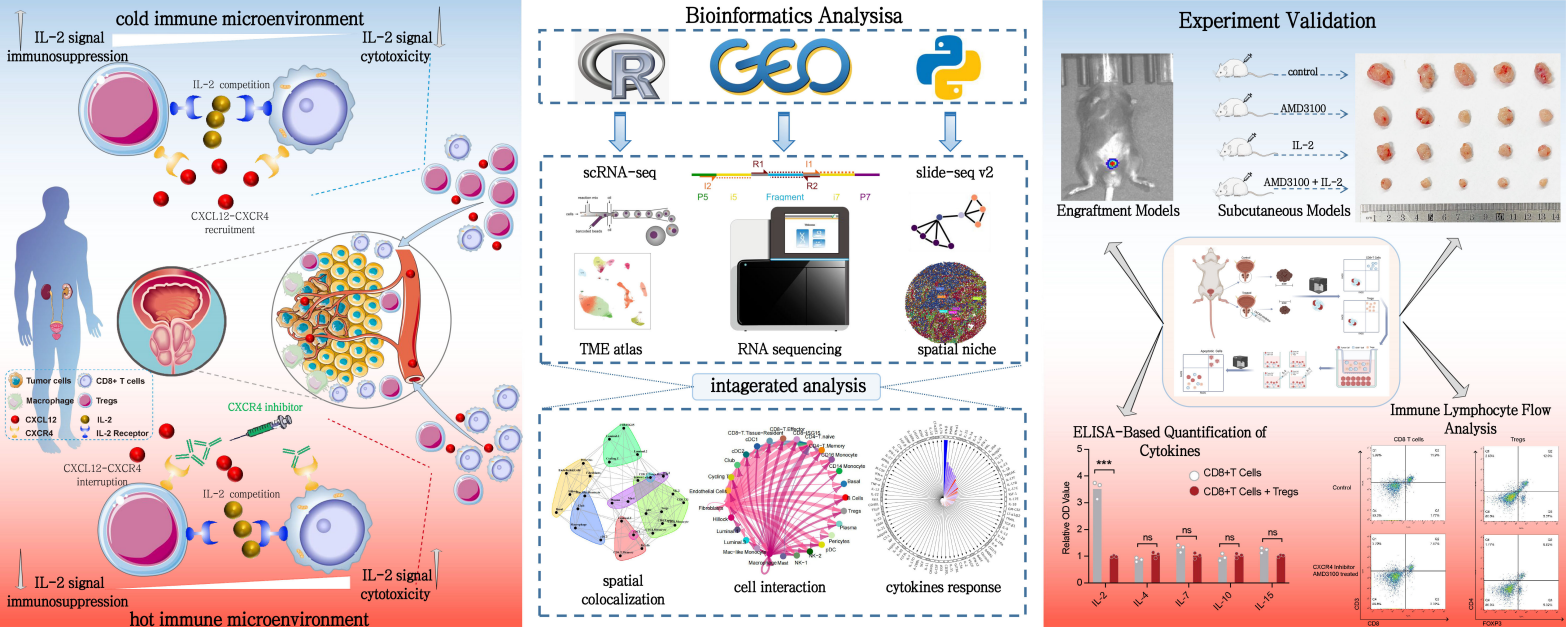
Integrated study design and methodology for prostate cancer immune microenvironment analysis. This figure provides a comprehensive view of the study design and methodology for analyzing the immune microenvironment in PCa. The left panel illustrates the distinct immune landscapes, emphasizing the chemotactic role of the CXCL12/CXCR4 axis in recruiting CD8+ T cells and Tregs within the TME. Disruption of this axis is shown to remodel the immune microenvironment and restore CD8+ T cell function. The central panel outlines the bioinformatics workflow, integrating single-cell and spatial transcriptomics to analyze cell abundance, polarization states, and spatial interactions. Spatial deconvolution and colocalization analyses reveal key cellular interactions. The right panel depicts experimental validation using *in vivo* and *in vitro* models, highlighting the efficacy of CXCR4 inhibitor and IL-2 treatments in reversing immune dysfunction and enhancing antitumor responses.

**Figure 2:**
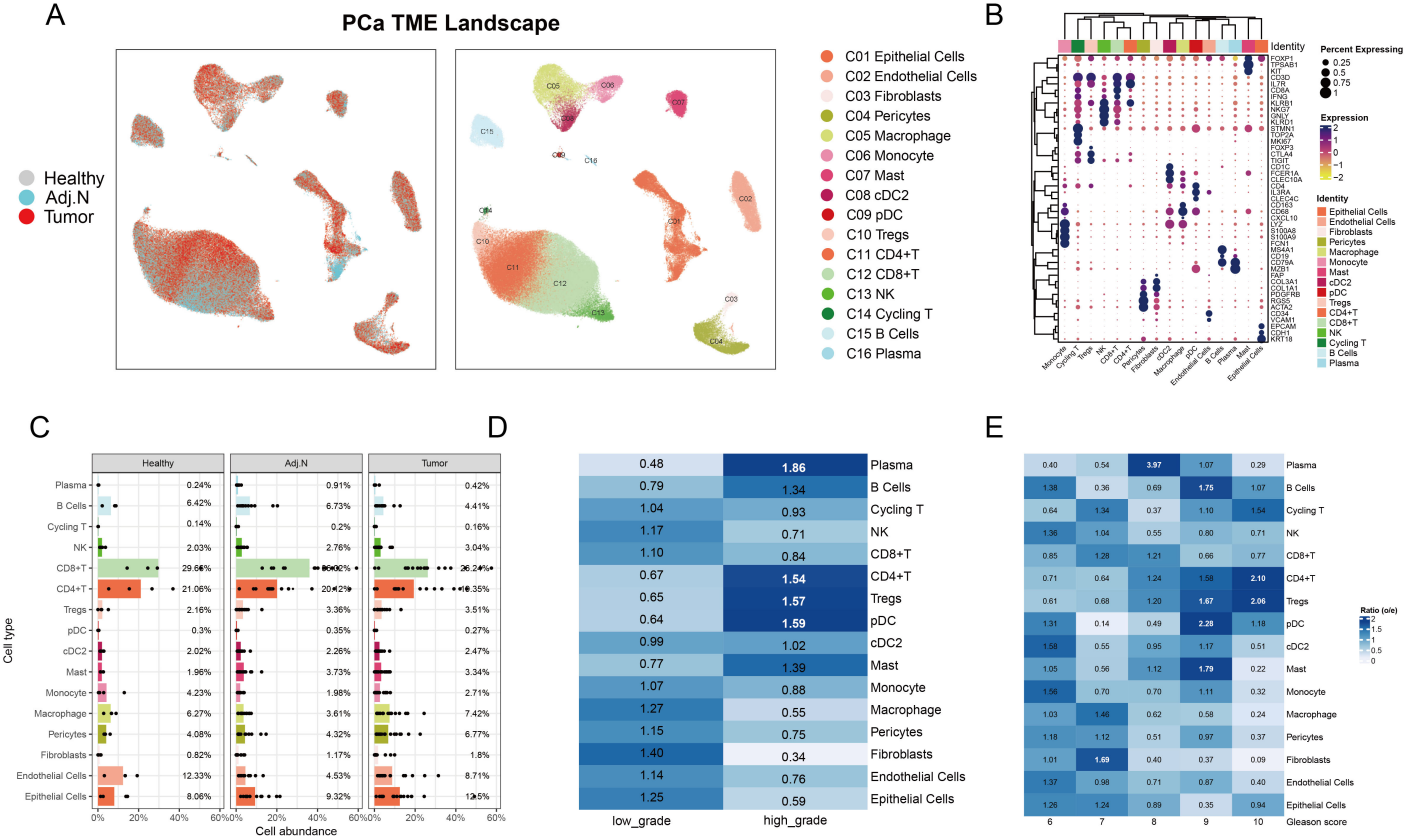
Characterization of the tumor microenvironment in prostate cancer at the single-cell level. **(A)** The UMAP plot illustrates the integration and visualization of single-cell data, depicting the data source on the left and cell annotations on the right for prostate cancer. **(B)** The dot plot displays marker gene expression for each cell type, where dot color indicates the level of gene expression and dot size corresponds to the percentage of cells expressing each gene. **(C)** The bar plot presents the average proportion of each cell type calculated from the various sample sources. **(D)** Analysis of observed-to-expected (O/E) ratio reveals cellular preferences in low-grade versus high-grade tumor samples; a ratio greater than 1 indicates a preference for that specific tissue type. **(E)** O/E ratio analysis across different Gleason score groups highlighted variations in cellular distribution associated with tumor aggressiveness.

The study design and overarching methodology are depicted in **Figure 1**. Following stringent quality control measures, we retained a total of 147,227 single cells from 4 healthy samples, 14 adjacent normal tissues, and 18 localized tumor samples for subsequent analysis. We constructed a comprehensive PCa atlas that encompasses 16 major cell types, highlighting the substantial presence of immune cells within the TME that retained their biological significance (**Figure 2A**). The marker genes pertinent to each cell type are illustrated in the heatmap, while hierarchical clustering is employed to characterize these cell populations (**Figure 2B**). To elucidate the cellular composition of the PCa TME, we observed significant heterogeneity across the cell proportions in each sample (**Supplementary Figure S1A**). We calculated the mean cell proportions and the observed-to-expected (O/E) ratio across different groups to gain insights into the biological implications of the cellular composition. The cell proportion analysis quantifies relative variations in cellular composition within the TME, while the O/E ratio provides a statistically robust measurement of intergroup differences through normalization against expected biological distributions. By integrating these complementary approaches - assessing relative abundance changes through cellular proportion and evaluating statistical deviations via O/E ratio - we established a dual-verification framework to identify pivotal cell populations that demonstrate both quantitative alterations and significant differential patterns in PCa TME. Notably, we discovered a striking phenomenon: CD4+ and CD8+ T cells were significantly reduced in tumor tissues, whereas epithelial cells, Tregs, and macrophages exhibited increased abundance (**Figure 2C**). Furthermore, the O/E ratio indicated that high-grade tumors presented an elevated abundance of CD4+ T cells and Tregs, alongside a decreased presence of CD8+ T cells and macrophages (**Figure 2D**; **Supplementary Figure S1B**). In tumor tissues, a clear pattern emerged where decreased abundance of CD8+ T cells and macrophages was accompanied by a marked increase in Tregs, as evaluated in relation to Gleason scores (**Figure 2E**). Gleason scores, which signify the degree of tumor malignancy, showed an association between higher malignancy and an immunosuppressive shift in the TME. Specifically, tumors with elevated Gleason scores exhibited an enrichment of Tregs, known for their role in dampening immune responses, and a decline in CD8+ T cells, which are critical for antitumor immunity. This immunosuppressive cellular composition may help elucidate why PCa is often categorized as an immune “cold” tumor, highlighting the impact of tumor aggressiveness on immune dynamics.

### 3.2 Identification of epithelial subtypes and malignant cells

**Figure 3:**
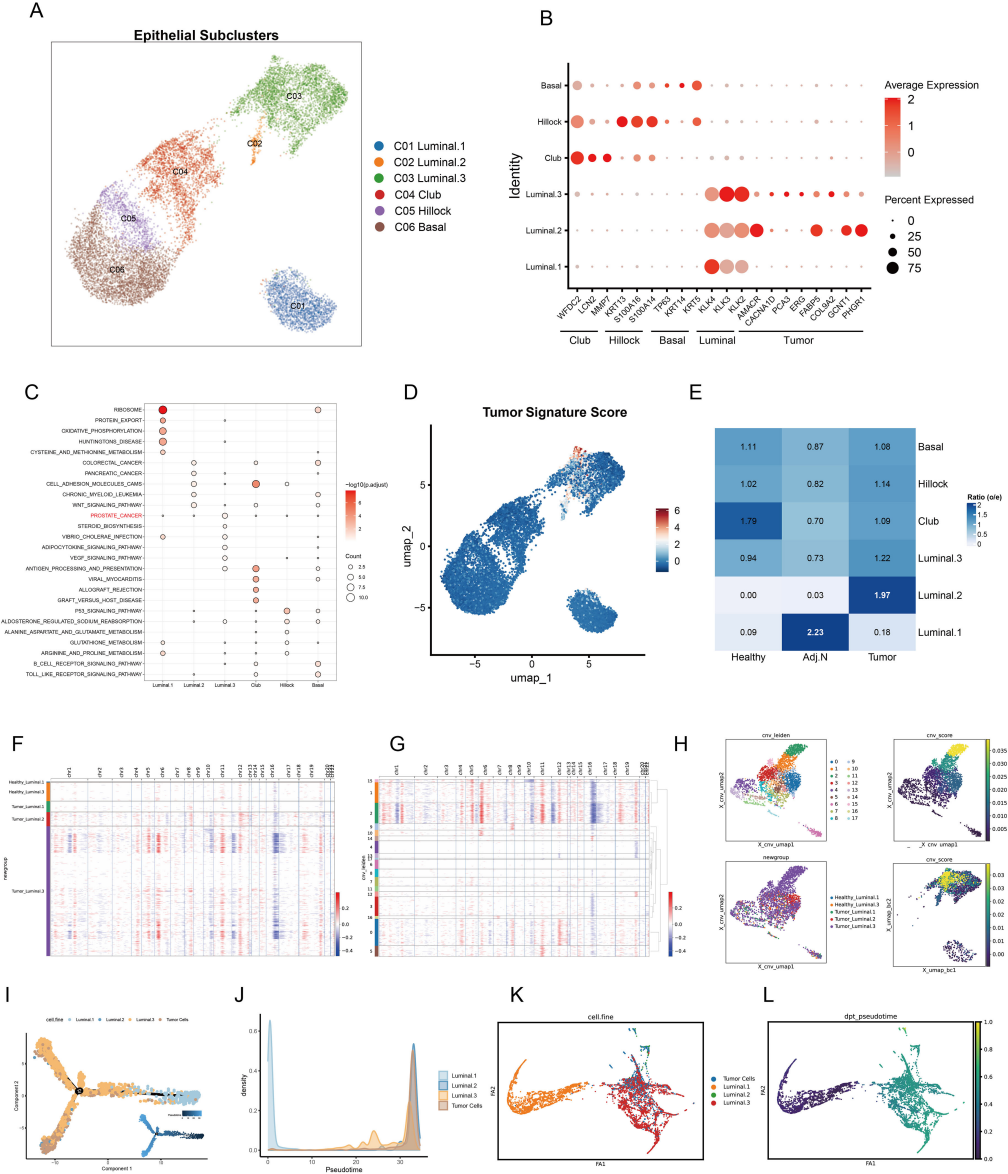
Identification of epithelial subpopulations and malignant cells in prostate cancer. **(A)** The UMAP embedding illustrates the identification of six distinct epithelial subtypes within the tumor microenvironment. **(B)** Marker gene expression and tumor signatures for each epithelial subtype are presented, highlighting key differentiating characteristics. **(C)** Pathway enrichment analysis of differentially expressed genes for each subtype is shown; dot color indicates the adjusted p value, while dot size represents the number of genes associated with each pathway. **(D)** Tumor signature scores calculated for each epithelial cell are displayed, providing insight into the malignant potential of these cells. **(E)** O/E ratio analysis across various data sources, including healthy, adjacent normal, and tumor tissues, reveals cellular distribution patterns. **(F, G)** A heatmap illustrates the copy number variation (CNV) in luminal cells derived from both healthy and tumor tissues, with CNV variations categorized by different Leiden clusters. Red indicates copy number amplification, whereas blue signifies copy number deletion. **(H)** The UMAP plot depicts CNV-based Leiden clustering, highlighting the relationships between CNV scores of individual luminal cells. **(I)** Cell trajectory analysis elucidates differentiation pseudotime and directional dynamics among various luminal subtypes and tumor cells. **(J)** The abundance of each cell type is tracked alongside pseudotime progression. **(K, L)** Partition-based graph abstraction analysis captures the cell embedding and pseudotime trajectories of luminal and tumor cells, facilitating an understanding of their developmental ways.

Unsupervised clustering analysis revealed four distinct epithelial subtypes: basal, hillock, club, and luminal cells. As depicted in previous research, luminal cells exhibit distinct functional characteristics and varying tumorigenic potentials. These cells play a crucial role in the progression and heterogeneity of prostate cancer, with evidence suggesting that different subsets of luminal cells contribute uniquely to tumor initiation, growth, and metastasis (24). Notably, the luminal cell population exhibited significant heterogeneity, leading to the classification of three subclusters (**Figure 3A**). The dot plot illustrated the marker genes associated with each subtype, revealing that luminal subclusters 2 and 3 expressed tumor-related genes (**Figure 3B**). Subsequent pathway enrichment analysis of DEGs confirmed functional variations across epithelial subtypes, with luminal subcluster 3 primarily enriched in prostate cancer pathways (**Figure 3C**). Additionally, the tumor signature score corroborated these findings (**Figure 3D**). The O/E ratio indicated a preference for luminal subclusters 2 and 3 within tumor tissue (**Figure 3E**). Consequently, we subjected all luminal cells from the tumor group to CNV and clustering analyses (**Figure 3F**). Clusters 0, 1, 2, 5, and 15 displayed elevated CNV scores and were identified as tumor cells (**Figures 3G, H**). To further elucidate the differentiation relationships among each subtype, trajectory analysis was conducted. Luminal subtype 1 was identified as the initial state of differentiation, while tumor cells exhibited high abundance in the terminal state (**Figures 3I, J**). Furthermore, Partition-based graph abstraction (PAGA) was employed to validate these results (**Figures 3K, L**).

### 3.3 The immunosuppressive state of lymphocyte subtypes in the prostate cancer

**Figure 4:**
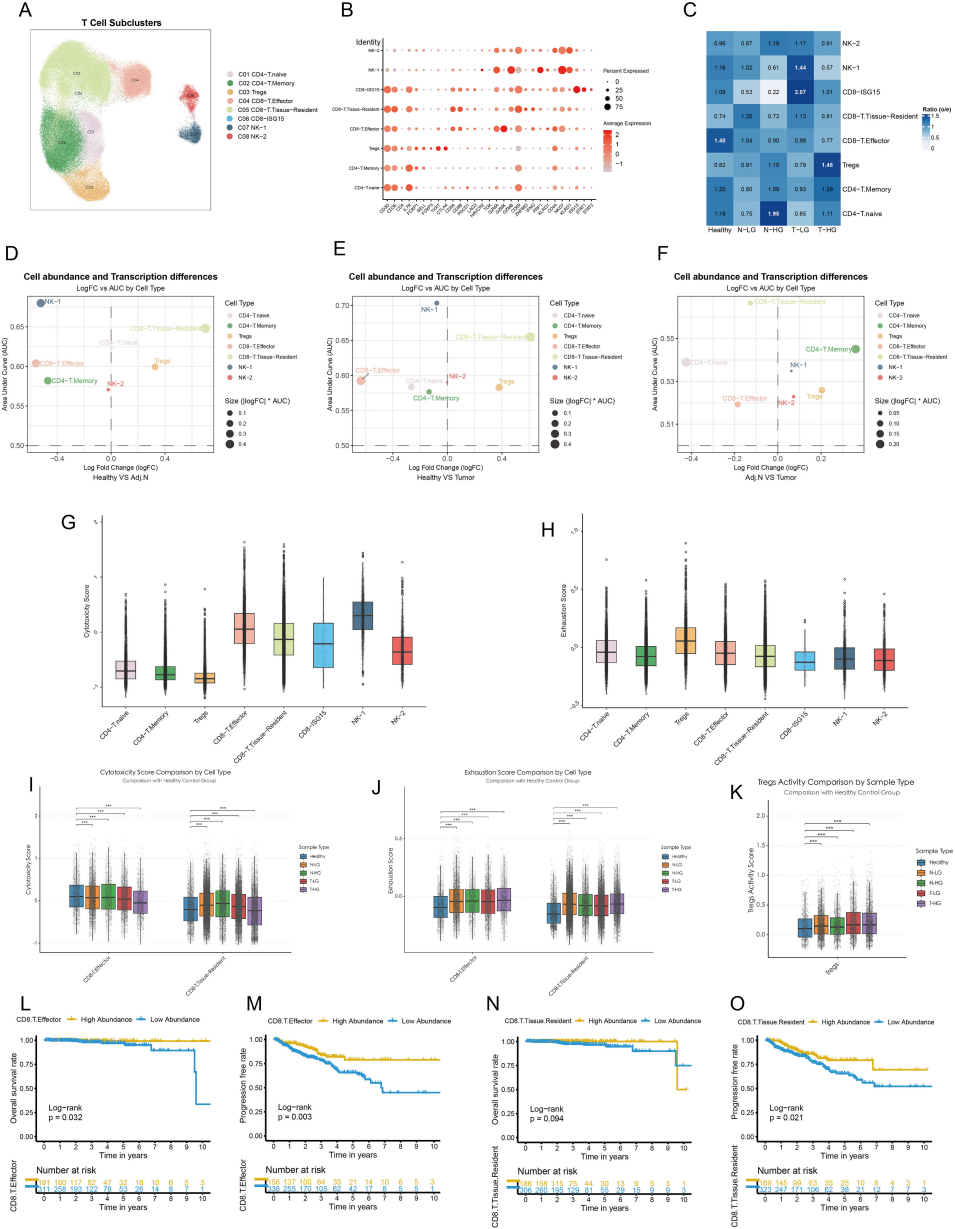
Dysfunctional CD8+ T cells and elevated treg activity in the prostate Tumor Immune Microenvironment. **(A)** Major lymphocyte subtypes identified within the tumor immune microenvironment are visualized using a UMAP plot. **(B)** Cell markers utilized to categorize these lymphocyte subtypes are presented. **(C)** Observed-to-expected (O/E) analysis reveals cellular tissue preferences among healthy, low-grade or high-grade adjacent normal, and tumor tissues. **(D–F)** Integrated MiloR and Augur analyses elucidate cell abundance and transcriptional differences among subpopulations across healthy, adjacent normal, and tumor samples. **(G, H)** Cytotoxicity activity and exhaustion scores are calculated based on gene signatures for each cell type, providing insights into their functional status. **(I, J)** Cytotoxicity and exhaustion scores of Tregs, CD8+ effector T cells, and CD8+ tissue-resident T cells are compared across different tissue sources. **(K)** Treg activity scores are calculated for various tissue sources, highlighting variations in Treg function. **(L, M)** Kaplan-Meier survival curves illustrate the prognostic impact of CD8+ effector T cells on overall survival and progression-free intervals. **(N, O)** Kaplan-Meier survival curves depict the prognostic significance of CD8+ tissue-resident T cells on overall survival and progression-free intervals. The patients were categorized based on the optimal survival cutoff value. Statistical significance was assessed using appropriate tests, where ***p < 0.001 and ns denotes not significant.

The TME of PCa was characterized by a substantial diversity of lymphocyte subtypes, specifically eight distinct categories, each exhibiting significant transcriptional characteristics (**Figures 4A, B**). The analysis of the O/E ratio provided insights into the potential mechanisms underlying the immunosuppressive nature of the TME in PCa. Notably, CD8+ effector T cells were significantly enriched in healthy tissue and displayed high abundance in high-grade adjacent normal and tumor tissues compared to their corresponding counterparts. In contrast, CD8+ tissue-resident T cells constituted a lower population in healthy tissue but exhibited similar trends as CD8+ effector T cells within higher-grade adjacent normal and tumor samples. Intriguingly, Tregs demonstrated a contrasting profile, with high abundance in high-grade tumors and adjacent normal tissues, while their presence was significantly diminished in healthy tissue (**Figure 4C**; **Supplementary Figures S2A, B**). To comprehensively evaluate these findings, we employed an unbiased algorithm for calculating abundance and transcriptional variations among lymphocytes across healthy, adjacent normal, and tumor tissues. Compared to healthy tissues, both CD8+ effector and tissue-resident T cells, as well as Tregs, revealed notable transcriptional disparities in adjacent normal and tumor tissues (**Figures 4D, E**; **Supplementary Figures S2C, D**). However, there were no significant differences in the transcriptional profiles of these cell types when comparing tumor tissues and adjacent regions (**Figure 4C**; **Supplementary Figure S2E**). Subsequent spatial differential abundance analysis illustrated a gradual decrease in CD8+ effector T cells correlating with tumor progression, alongside an increase in tissue-resident CD8+ T cells primarily localized in tumor and adjacent areas (**Figures 4D–F**; **Supplementary Figure S2F–H**). Notably, CD8+ T cells expressing high levels of ISG15 exhibited minimal transcriptional and population variation.

Other immune cell types did not exhibit distinguishable patterns. To further investigate the functional roles of the pivotal subtypes outlined, we assessed cytotoxicity scores, revealing that CD8+ effector T cells exhibited the highest cytotoxicity, whereas CD8+ISG15 T cells showed the lowest levels of cytotoxic activity within the CD8+ T cell population (**Figure 4G**). Additionally, Tregs displayed significant signs of functional exhaustion (**Figure 4H**). We observed a reduction in the cytotoxic functions of CD8+ effector T cells correlated with migration to malignant tissues and tumor progression. Conversely, CD8+ tissue-resident T cells exhibited elevated cytotoxicity scores in adjacent normal tissues, which decreased within tumor tissues (**Figure 4I**). Furthermore, all pivotal subpopulations demonstrated functional exhaustion associated with tumorigenesis (**Figure 4J**). Importantly, within tumor tissues, Tregs displayed pronounced activity (**Figure 4K**). Besides, Expression of key signature genes associated with cytotoxicity, exhaustion, and Treg activity across lymphocyte subclusters were presented in **Supplementary Figures S3A–C**. Survival analysis indicated that PCa patients with high abundance of CD8+ effector T cells experienced longer survival times and extended disease-free intervals (**Figures 4L, M**). Although the abundance of CD8+ resident T cells did not significantly affect overall survival, it did impact the recurrence time of tumors (**Figures 4L–O**). The abundance of Tregs did not impose a poorer prognosis in PCa patients (**Supplementary Figure S2I, J**). In other words, Tregs may not directly influence the prognosis of PCa. Instead, they might mediate the abundance and functional variation of CD8+ T cells, thereby contributing to the modulation of the immunosuppressive TME and indirectly affecting patient outcomes.

### 3.4 Guilt myeloid subcluster detection

**Figure 5:**
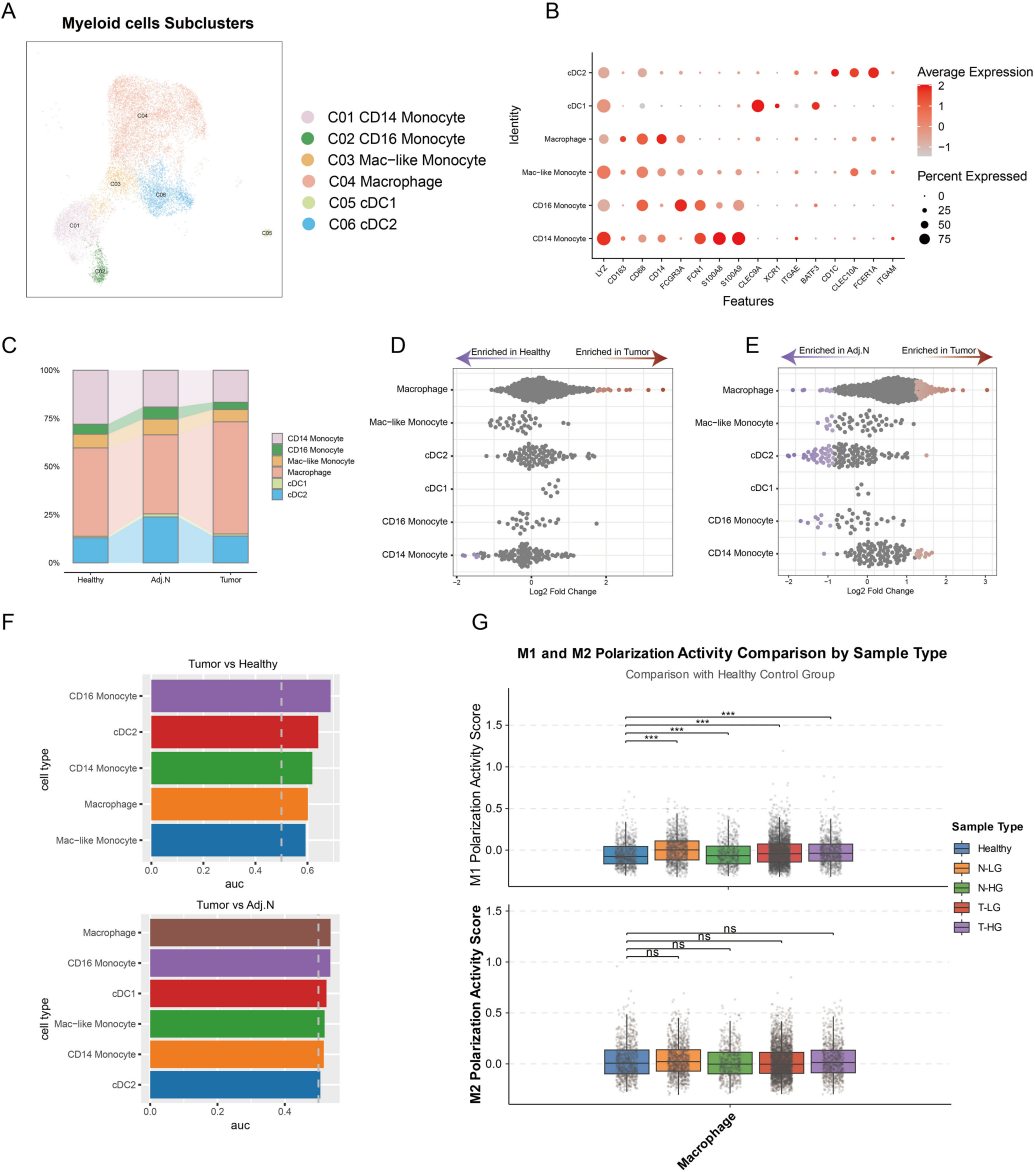
Identification of pivotal myeloid cell subtypes in prostate cancer. **(A)** Integrated embedding demonstrates detailed annotations of myeloid subclusters within the tumor microenvironment. **(B)** The dot plot presents key cell markers used for annotation of myeloid cell subtypes. **(C)** A bar plot reveals the cell population distributions of each myeloid subtype across different tissue groups. **(D, E)** Unbiased cellular abundance analysis identifies pivotal myeloid cell types by comparing tumor samples to healthy controls and adjacent normal tissues. **(F)** Transcriptomic disparity analysis highlights differences in myeloid subtypes across various data sources. **(G)** M1 and M2 polarization states are determined based on gene signatures identified across three distinct tissue sources. ***p < 0.001. ns, not significant.

To further investigate the mechanisms underlying the modulation of the immunosuppressive TME, all myeloid cells underwent comprehensive clustering and annotation. Six subpopulations were identified and annotated based on classical marker genes (**Figures 5A, B**). The analysis of cell proportions revealed a significant increase in macrophages within the tumor group compared to adjacent normal and healthy tissues (**Figure 5C**). Additionally, differential abundance analysis corroborated these findings based on the unbiased embedding, indicating a marked elevation of macrophages in tumor tissue relative to both adjacent normal and healthy tissues (**Figures 5D, E**). Additionally, analysis using the ‘Augur’ algorithm revealed pronounced transcriptional differences in macrophages when comparing tumor tissues with both healthy and adjacent normal tissues (**Figure 5F**). The polarization states of M1 and M2 macrophages were systematically analyzed across different tissue types, including healthy tissue, low-grade normal tissue, high-grade normal tissue, low-grade tumor tissue, and high-grade tumor tissue. The analysis revealed significant variations in M1 macrophage polarization in tumor-adjacent and tumor tissues compared with healthy tissues, suggesting that macrophages within the TME undergo polarization toward the M1 phenotype to exert anti-tumor functions (**Figure 5G**). In contrast, no substantial changes in M2 polarization were observed in tumor-related tissues. These findings underscore that macrophages in PCa not only exhibit an increase in cellular abundance but also undergo marked transcriptional reprogramming, driving their polarization toward an M1-like phenotype to mediate anti-tumor immunity. This highlights their critical regulatory role in the development and progression of PCa, potentially influencing other immune cell populations and contributing to the complexity of the immunosuppressive TME.

Therefore, it can be concluded that the immunosuppressive tumor microenvironment is not solely regulated by macrophages. Rather, they may mediate the influence of other immune cell types in shaping the immunosuppressive TME.

### 3.5 Cell niche and colocalization analysis in the spatial context

**Figure 6:**
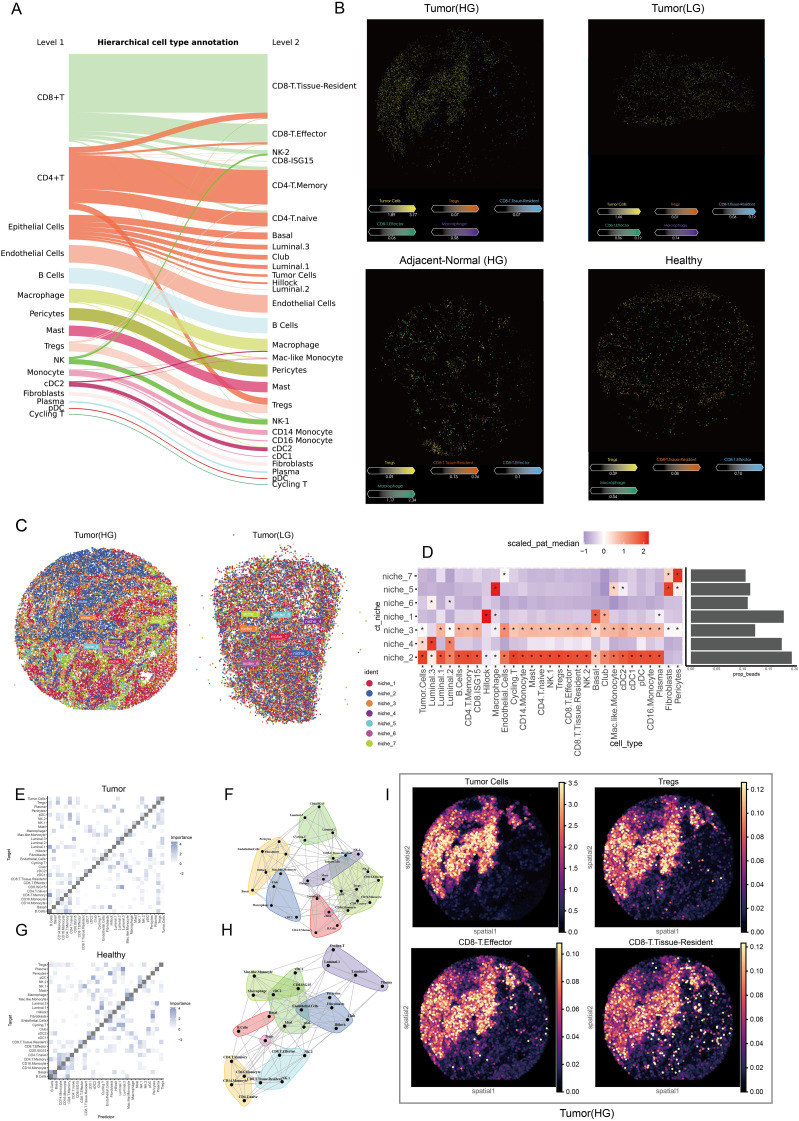
Spatial characteristics and relationships in prostate cancer. **(A)** The Sankey plot illustrates the refined cell annotation process, with level 2 annotations utilized for downstream analyses. **(B)** The slide presents pivotal cells identified at the single-cell level within the spatial context of low-grade and high-grade tumor tissues, high-grade adjacent normal tissues, and healthy tissues. Colors correspond to different cell types, while the color bar indicates the abundance of each cell type in the beads. **(C)** Cell niche analysis reveals potential cell communities by integrating spatial transcriptomic data from low-grade and high-grade tumors. **(D)** A heatmap displays the cellular composition of each niche, while a bar plot illustrates the proportion of each niche; colors represent abundance, and ‘*’ indicates significant enrichment. **(E)** The heatmap shows the localized relationships between predictor cells and target cells within the spatial context of tumor samples. **(F)** The pattern plot depicts spatial colocalization relationships and connectivity among various cell types within the tumor microenvironment. **(G)** A heatmap demonstrates the localized relationships between predictor cells and target cells in the spatial context of healthy samples. **(H)** The pattern plot illustrates spatial colocalization relationships and connectivity across various cell types in healthy tissue. **(I)** The spatial density plot shows the spatial distribution of tumor cells, Tregs, CD8+ T effector, and CD8+ T tissue-resident cells, highlighting their colocalization and spatial coordination within the tumor microenvironment.

The Sankey plot illustrated the refined process of cell annotation, while Level 2 was employed to conduct spatial deconvolution analysis (**Figure 6A**). Key cellular populations were identified within the spatial context across multiple tissue sources (**Figure 6B**). Tumor cells exhibited complex interactions with immune cells, reflecting intricate relationships at specific spatial locations. Consequently, samples of low and high grades were integrated to investigate the cellular niches associated with each population. Seven distinct niches were delineated within the TME (**Figure 6C**). The heatmap revealed significant variations in cellular composition across different niches, highlighting sophisticated interactions between tumor cells and CD8+ effector T cells, CD8+ tissue-resident T cells, Tregs, and macrophages specifically in niches 2 and 3 (**Figure 6D**). Furthermore, spatial colocalization analysis was conducted to elucidate the differences in cellular spatial relationships between healthy and tumor tissues. In the tumor tissue, we observed that tumor cells, CD8+ effector T cells, and Tregs exhibited close juxtapositional colocalization. Although CD8+ tissue-resident T cells were closely associated with Tregs, they were isolated from the aforementioned juxtapositional region (**Figures 6E, F**). In contrast, Tregs were not located in proximity to CD8+ effectors or tissue-resident T cells in healthy tissue (**Figures 6G, H**). In **Figure 6I**, the spatial colocalization patterns in tumor tissues are further detailed, highlighting the proximity of tumor cells, CD8+ effector T cells, and Tregs. Because the slide-seq V2 platform does not achieve single-cell resolution (each bead typically contains multiple cells), the data inherently aggregates adjacent cells that are in close physical proximity. This spatial proximity naturally leads to similar patterns in the distribution of tumor cells and immune subpopulations. This close arrangement contrasts with the separation of CD8+ tissue-resident T cells from the main juxtapositional region. The distinct colocalization of these immune cells with tumor cells supports the hypothesis that such spatial arrangements may facilitate tumor progression by fostering an immunosuppressive microenvironment.

### 3.6 CXCL12-CXCR4 ligand-receptor interaction mediates Treg-induced exhaustion of CD8+ T cells and potentiates antitumor immunity

**Figure 7:**
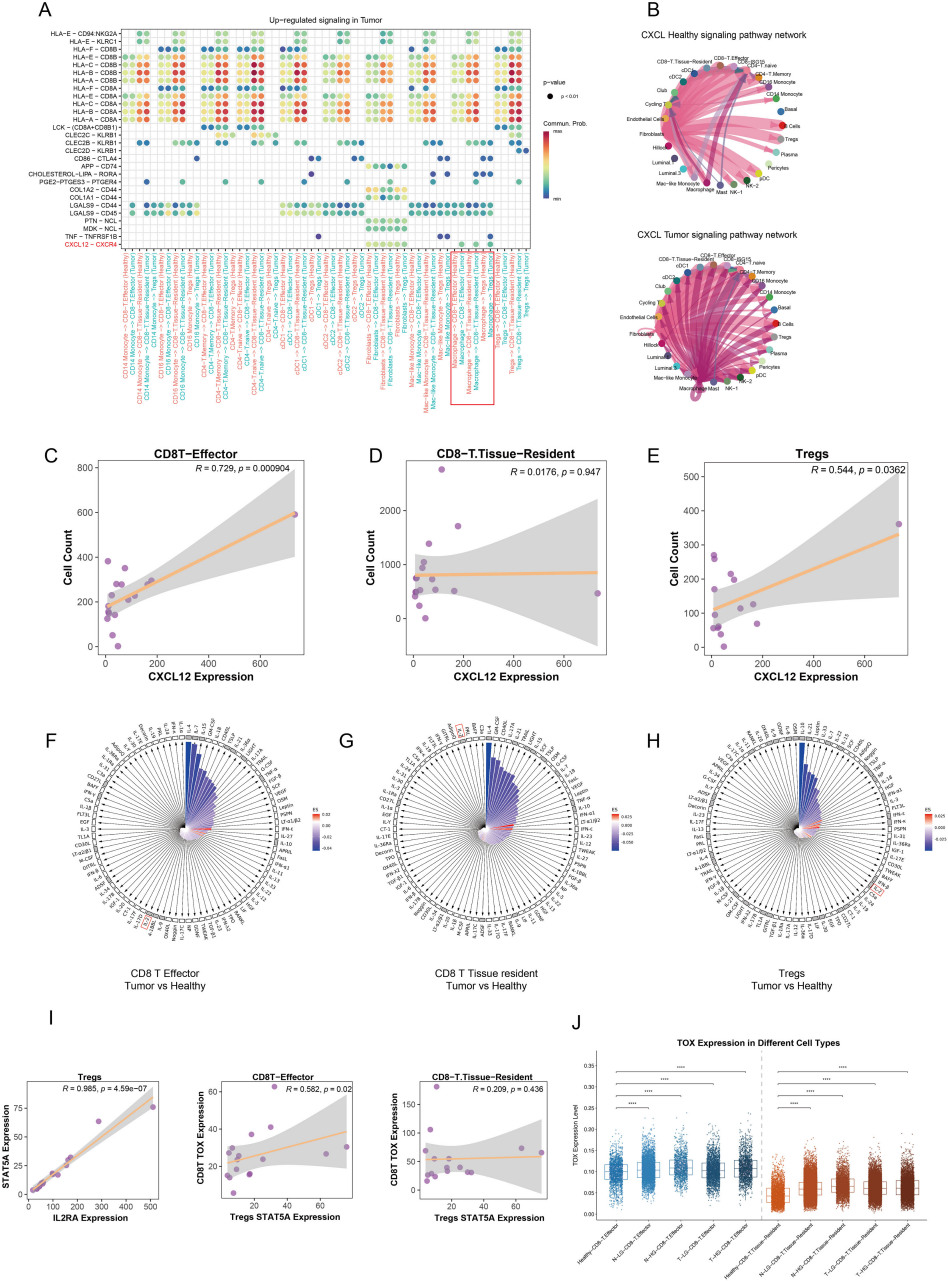
Modulation of the immunosuppressive microenvironment in prostate cancer. **(A)** The dot plot illustrates the upregulated signaling pathways in the tumor group across various immune cell types, with color intensity representing the strength of communication between cells. **(B)** The circular plot depicts the CXCL signaling pathway network among different cell types, comparing healthy and tumor groups. **(C–E)** Correlation analyses reveal the potential relationships between CXCL12 gene expression and the abundance of CD8+ effector T cells, CD8+ tissue-resident T cells, and Tregs in each tumor sample. **(F–H)** The polar diagram displays the immune cell response states of CD8+ effector T cells, CD8+ tissue-resident T cells, and Tregs under stimulation by 86 cytokines within the tumor group. The small black grid indicates receptor gene expression, while white squares represent non-expressed receptors. The lengths of the bars correspond to p values, with longer bars indicating higher significance. Bar colors reflect estimated scores, where more intense red indicates a closer similarity in response states. **(I)** Pseudobulk analysis demonstrates the correlation between STAT5A and IL2RA expression levels in Tregs at the sample level. Additionally, the relationship between STAT5A expression in Tregs and TOX gene expression in both CD8+ effector and tissue-resident T cells is shown. **(J)** A comparison of TOX expression levels in CD8+ effector and tissue-resident T cells across various tissue types is presented. ****p < 0.0001.

After establishing the variation in cell populations and their spatial colocalization, we conducted a cell communication analysis to investigate interactions among different cell types. DEA was performed on L-R gene expression profiles at the single-cell level, revealing notable insights regarding the CXCL12-CXCR4 L-R pair. This interaction significantly increased between macrophages and CD8+ effector T cells, tissue-resident T cells, and Tregs within the TME (**Figures 7A, B**). Although fibroblasts expressed this L-R signal, they were not found in the same cellular niches as the other cell types in this spatial context (**Figure 6D**). The validated spatial relationship between macrophages and key lymphocyte populations underlines the importance of macrophage-derived CXCL12 in recruiting CD8+ effector cells and Tregs from blood vessels or lymph nodes (**Figures 7C, E**). Importantly, CD8+ tissue-resident cells, which function as memory cells residing *in situ*, did not exhibit significant population changes despite elevated macrophage’s CXCL12 expression (**Figure 7D**). CD8+ effector cells and Tregs are actively recruited into the PCa TME by macrophages, with macrophages primarily displaying an M1 polarization state. This recruitment serves to facilitate anti-tumor immune responses while concurrently reshaping the immune landscape within the tumor. Macrophage-derived CXCL12 appears to play a pivotal role in this process, enabling the trafficking of CD8+ effector cells and Tregs from peripheral blood vessels or lymph nodes into the TME. Notably, while CD8+ effector cells contribute to anti-tumor immunity, the concurrent presence of Tregs may represent a balancing mechanism in the immune response, highlighting the dynamic and complex interplay between immune cell populations in shaping the immunosuppressive and immunoreactive characteristics of the TME. To further elucidate the mechanisms underlying the functional exhaustion of CD8+ T cells, we employed IREA, utilizing an immune dictionary to assess cytokine effects. IL-2 is a critical cytokine that plays a significant role in mediating antitumor and antiviral responses, influencing the effector and memory functions of CD8+ T cells (25). Recent studies have indicated that Tregs can deplete and sequester IL-2, thereby suppressing the activation and cytotoxicity of CD8+ T cells (26). CD8+ effector and tissue-resident T cells expressed numerous IL-related receptor genes; however, they lacked stimulation from IL-related cytokines such as IL-2, IL-4, IL-7, and IL-15 when comparing response states between tumor and healthy groups (**Figures 7F, G**). In contrast, Tregs exhibited upregulated expression of receptor genes and a pronounced response to interferon (IFN) and IL-2 cytokines (**Figure 7H**). There is compelling evidence that Tregs and CD8+ effector T cells—not CD8+ tissue-resident T cells—are located in close proximity within the TME, allowing for mutual interaction (**Figures 6D–F**).

Based on these findings, we propose a hypothesis suggesting that the functional exhaustion of CD8+ effector T cells results from Tregs fully engaging IL-2 within the juxta-TME. CD8+ tissue-resident T cells remain unaffected by Tregs due to their distant spatial positioning. To further explore this potential mechanism, recent studies have confirmed that Tregs compete for intratumoral IL-2, mediating IL-2/STAT5 signaling to inhibit the activation of tumor-infiltrating CD8+ T cells and induce exhaustion through the upregulation of thymocyte selection-associated high mobility group box protein (TOX) (27). Consequently, we constructed pseudobulk data to decipher the relationships among IL-2 response states, STAT5 activation in Tregs, and TOX expression in CD8+ effector and tissue-resident T cells within tumor samples. Our results indicated a significant positive correlation between IL-2 receptor A (IL2RA) gene expression and STAT5A expression in Tregs. Furthermore, the TOX expression in CD8+ effector T cells was induced by Treg-mediated IL-2/STAT5 activation, while CD8+ tissue-resident T cells showed no corresponding changes in tumor samples (**Figure 7I**). Differential expression analysis at the single-cell level revealed that TOX expression was significantly elevated in both CD8+ effector and tissue-resident T cells in tumor and adjacent normal tissues compared to healthy tissues. Notably, CD8+ effector T cells exhibited higher TOX levels than CD8+ tissue-resident T cells (**Figure 7J**). This observation indirectly supports the hypothesis that Treg-mediated IL-2/STAT5 signaling contributes to TOX-driven exhaustion of CD8+ effector T cells. To further substantiate this hypothesis, classical CD8+ T cell exhaustion and immune checkpoint genes, PDCD1 and CD274, were examined to assess the state of the cells of interest. Both genes were noticeably upregulated in CD8+ effector and tissue-resident T cells within the tumor and adjacent normal tissues (**Supplementary Figures S4B–D**). Intriguingly, the increased expression of PDCD1 and CD274 positively correlated with the activation of the Tregs’ IL-2/STAT5 signaling, a relationship not observed in CD8+ tissue-resident T cells (**Supplementary Figures S4A–C**).

In summary, our findings indicate that macrophages mediate CXCL12-CXCR4 ligand-receptor interactions to recruit Tregs and CD8+ effector T cells, leading to functional exhaustion of CD8+ effector T cells through competition for intratumoral IL-2 by Tregs in prostate cancer.

### 3.7 Disruption of the CXCL12-CXCR4 axis restores CD8+ T cell cytotoxicity and enhances antitumor immunity

**Figure 8:**
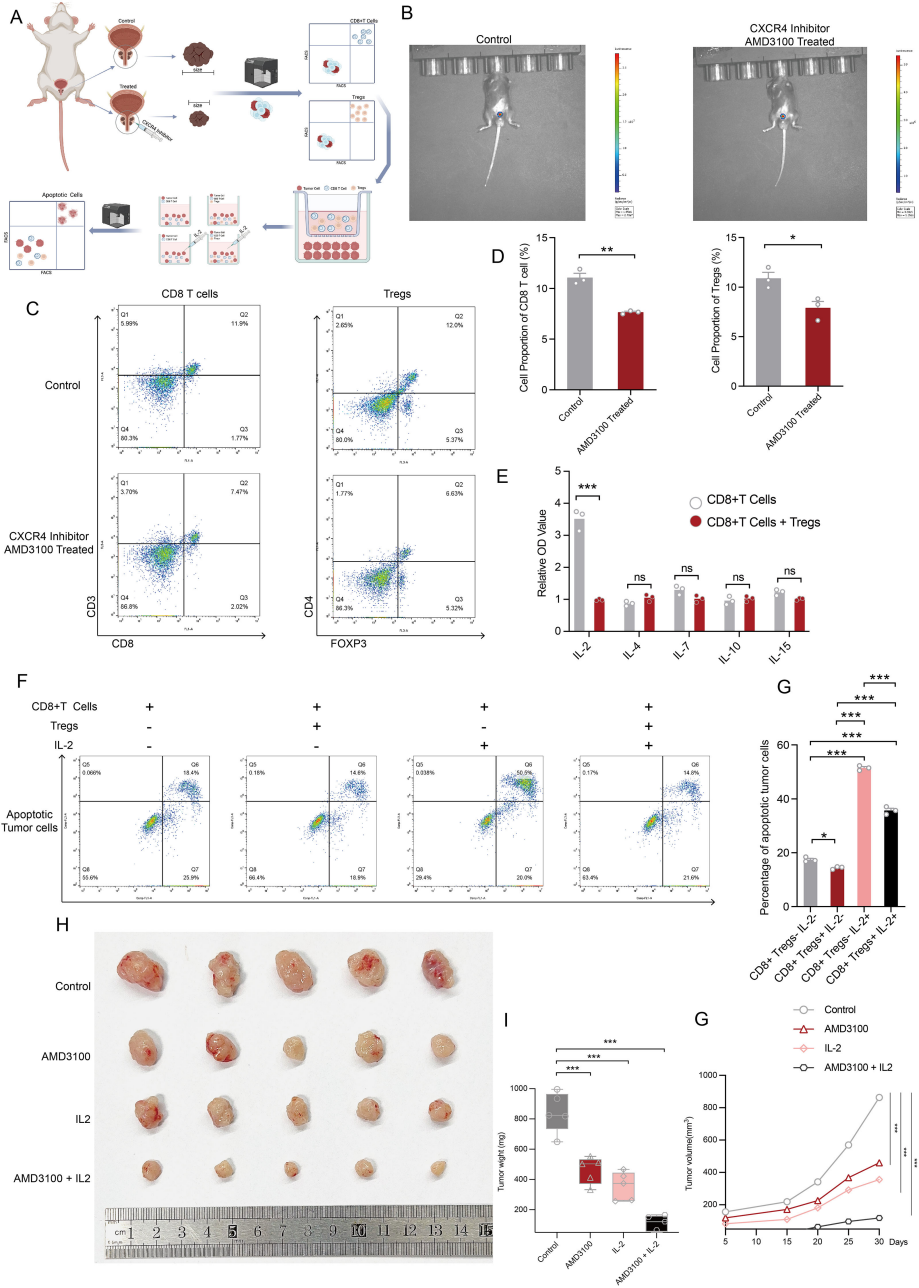
Disruption of CXCL12/CXCR4 signaling reverses the dysfunctional state of CD8+ T cells. **(A)** The flowchart outlines the experimental design and procedures for both *in vivo* and *in vitro* assays. This figure was generated using BioRender. **(B)** *In vivo* imaging demonstrates the size of prostate tumors treated with PBS compared to those treated with an CXCR4 inhibitor inhibitor. **(C)** Flow cytometry results depict the abundance of CD8+ T cells and Tregs after CXCR4 inhibitor treatment. **(D)** The bar plot presents disparities in the abundance of CD8+ T cells and Tregs following CXCR4 inhibitor treatment. **(E)** ELISA assay quantifying protein levels of IL-2, IL-4, IL-7, IL-10, and IL-15 in the microenvironment of *in vitro* cultures of CD8+ T cells, as well as co-cultures of CD8+ T cells and Tregs. **(F)** Apoptotic flow detection illustrates the cytotoxic effects of CD8+ T cells on co-cultured versus non-co-cultured Tregs, with or without IL-2 supplementation. **(G)** The bar plot displays the percentage of apoptotic tumor cells across four experimental conditions. **(H)** Tumors from each condition were harvested and photographed, showcasing differences across the four treatment groups. **(I)** A comparative analysis of tumor wight among the four treatment groups is presented. **(J)** Tumor volume measurements over time across different treatment groups. Statistical significance was assessed using appropriate tests, where *p < 0.05, **p < 0.01, ***p < 0.001 and ns denotes not significant.

To further validate our conclusions derived from single-cell and spatial TME analyses, we conducted *in vivo* and *in vitro* experiments to strengthen our findings. The experimental design and procedures are illustrated in **Figure 8A**. Following *in situ* tumorigenesis in mice, we administered an CXCR4 inhibitor drug via injection into the prostate, resulting in a significant reduction in tumor size in the treated group compared to the control group, as indicated by fluorescence intensity measurements (**Figure 8B**). Subsequently, tumor tissues were dissociated and analyzed using flow cytometry to assess the population dynamics of Tregs and CD8+ T cells. Our results demonstrated a significant reduction in both Tregs and CD8+ T cells within the tumor microenvironment following CXCR4 inhibitor treatment (**Figures 8C, D**). ELISA assays within the co-culture system demonstrated that Tregs competitively deplete IL-2 in conjunction with CD8+ T cells, thereby attenuating the immune activation of CD8+ T cells within the tumor microenvironment. Notably, levels of other cytokines, including IL-4, IL-7, IL-10, and IL-15, remained consistent between the Tregs co-culture and CD8+ T cells-alone groups (**Figure 8E**). Subsequently, We then performed co-culture assays to investigate the immunosuppressive function of Tregs and their role in IL-2 depletion, which contributes to the diminished cytotoxic function of CD8+ T cells. Three variables across four experimental groups thoroughly elucidated that Tregs suppress the cytotoxic activity of CD8+ T cells, and that IL-2 administration can reverse this exhaustion. In comparison with Group 1, which consisted solely of tumor cells and CD8+ T cells, CD8+ T cells in Group 3 exhibited significantly enhanced cytotoxicity, effectively eliminating a larger number of tumor cells. In Group 2, where CD8+ T cells were co-cultured with Tregs, there was a noticeable reduction in cytotoxic function. Conversely, Group 4, which received supplemental IL-2 cytokines, showed a substantial recovery of CD8+ T cell functionality, though it remained lower than that observed in Group 3, indicating that Tregs largely sequestered available IL-2 (**Figures 8F, G**). Finally, we conducted *in vivo* experiments involving subcutaneous neoplasia to explore the impact of disrupting the CXCL12-CXCR4 axis on tumor progression in the mice model. Twenty mice were categorized into four groups: control, CXCR4 inhibitor treated, IL-2 treated, and a combination of CXCR4 inhibitor and IL-2 treatments, to simulate therapeutic effects *in vivo*. Notably, the untreated control group exhibited the largest tumor sizes among all groups. Both single-variable treatment groups displayed reduced tumor sizes compared to the control group, while the combined treatment group demonstrated the smallest tumors (**Figures 8H, I**). In parallel, groups treated with either CXCR4 inhibitor or IL-2 alone showed a marked inhibition of tumor proliferation compared to controls. The combination therapy group, however, exhibited the most pronounced therapeutic benefit (**Figure 8G**). In conclusion, our findings suggest that disruption of the CXCL12-CXCR4 axis abrogates Treg-mediated immunosuppression and restores CD8+ T cell functionality during prostate cancer progression.

## 4 Discussion

Traditional bulk transcriptome analysis has provided insights into gene expression modulation at the tissue level; however, it often falls short in elucidating cell composition, cellular states, heterogeneity, and the intricate gene regulatory networks at the cellular level (28). In this study, the immune microenvironment of localized prostate cancer was examined at single-cell and spatial levels, yielding a detailed and comprehensive understanding of the cellular community. Notably, a novel underlying cellular crosstalk was identified, wherein macrophages utilize the CXCL12/CXCR4 ligand-receptor pair to recruit Tregs and CD8+ effector T cells to aggregate in the TME. The spatial colocalization of tumor cells, Tregs, and CD8+ effector T cells was validated, highlighting the physical conditions that facilitate Tregs in exerting immunosuppressive regulation over CD8+ effector T cells, preventing them from effectively targeting tumor cells. Additionally, the molecular mechanism was elucidated, revealing that Tregs deplete IL-2, thereby suppressing activation and inducing exhaustion of CD8+ effector T cells.

Macrophages are vital immune cells within the TME that exhibit high plasticity, allowing them to polarize into distinct phenotypes in response to environmental signals. This polarization significantly influences tumor initiation, progression, invasion, metastasis, and immune evasion (29, 31). Tumor-associated macrophages (TAMs), shaped by tumor-derived antigens, often exhibit phenotypic transitions that promote immune evasion and tumor growth (30, 31). Our findings revealed a marked increase in macrophage infiltration within tumor regions compared to healthy prostate tissue, accompanied by transcriptional variations. Notably, in tumor-adjacent and tumor tissues, macrophages predominantly polarized toward an M1 phenotype rather than the immunosuppressive M2 subtype, suggesting that the prostate cancer TME primarily stimulates macrophages to act as immune regulators and exert anti-tumor effects. Unlike cytotoxic immune cells, macrophages lack direct tumor-killing ability. M1 macrophages, characterized by their pro-inflammatory nature, play a crucial role in immune surveillance by clearing pathogens and abnormal cells. However, excessive activation can lead to tissue damage. Conversely, M2 macrophages exhibit anti-inflammatory properties, aiding tissue repair and immune regulation. In the TME, M1 macrophages enhance immune responses, creating an inflammatory milieu favorable for tumor cell destruction. They also modulate the activity and function of immune cells to bolster anti-tumor immunity. In contrast, M2 macrophages secrete anti-inflammatory cytokines like IL-10, suppressing immune activation. They additionally recruit various immune and stromal cells through cytokine and chemokine secretion, fostering a microenvironment conducive to tumor growth and progression (32, 33). In the prostate cancer TME, macrophages primarily polarize toward the M1 state and utilize CXCL12/CXCR4 crosstalk to chemoattract peripheral CD8+ effector T cells, which target and kill cancer cells, thereby impeding tumor progression. Unfortunately, this cellular interaction simultaneously recruits Tregs into the tumor microenvironment, introducing immunosuppressive regulation. Spatial transcriptomic analysis revealed that CD8+ effector T cells, Tregs, and tumor cells are closely situated within the TME. This proximity strongly suggests that Tregs impair the tumor-killing activity of CD8+ effector T cells, leading to immune suppression and subsequent tumor progression. Furthermore, the CXCL12/CXCR4 crosstalk between macrophages and lymphocytes was significantly amplified in the tumor context, providing deeper insight into the complex interplay driving the TME dynamics.

The CXCL12-CXCR4 chemokine axis has garnered significant attention for its multifaceted roles in tumor progression, including its impact on cancer cell survival, metastasis, and immune cell trafficking. This axis has been implicated in various cancer types, such as prostate, breast, and lung cancers, where it drives cancer progression through mechanisms that enhance proliferation, migration, invasion, angiogenesis, and immunosuppression (34, 35). Activation of CXCL12/CXCR4 triggers downstream signaling pathways, including the PI3K/AKT/mTOR and MAPK/ERK cascades, which promote tumor cell proliferation and survival (36). Moreover, CXCL12 is highly expressed in metastatic target organs, such as the bone, liver, and lungs, where it establishes chemotactic gradients to guide CXCR4+ tumor cells to these sites. Importantly, CXCL12 also plays a pivotal role in recruiting immunosuppressive cells, such as Tregs and myeloid-derived suppressor cells, into the tumor microenvironment, thereby fostering immune evasion and resistance to chemotherapy (37). Recent studies have further demonstrated that this crosstalk promotes angiogenesis in PCa, facilitating tumor progression (38). Moreover, activation of the CXCL12/CXCR4 signaling pathway has been shown to engage GPCR pathways, contributing to bone metastasis in PCa (39).

Disruption of this axis using CXCR4 inhibitors, such as AMD3100, can mitigate tumor progression to some extent. AMD3100 (Plerixafor) is a selective CXCR4 chemokine receptor antagonist that blocks the interaction between CXCR4 and its natural ligand CXCL12, a critical axis involved in various pathological conditions, including HIV-1 infection, tumor progression, and metastasis. AMD3100’s mechanism of action has been linked to mobilizing hematopoietic stem cells, increasing circulating neutrophils, lymphocytes, and monocytes, reducing myeloid-derived suppressor cells, and enhancing cytotoxic T cell infiltration into tumors (40). In mesothelioma specifically, AMD3100 has been shown to modulate T cell migration and TAMs infiltration, effectively reversing the immunosuppressive TME and enhancing responses to immunotherapy (23). Furthermore, the CXCR4/CXCL12 axis plays a multifaceted role in cancer, including leukemic stem cell homing and signaling. The targeting of CXCR4 has emerged as a promising therapeutic strategy for both hematologic malignancies and solid tumors (41). The tumor immune microenvironment (TIME) encompasses various immune cells, cytokines, and tumor cells, with its cellular composition and dynamic changes in cell states critically influencing the functional balance between antitumor immune activity and tumor-promoting processes. These characteristics are closely tied to tumor progression, recurrence, and metastasis. Therefore, reprogramming the TIME is essential for enhancing the efficacy of immunotherapy (42). The advent of high-throughput spatial transcriptomics technology introduces a novel dimension by providing insights into the spatial architecture and physical proximity of elements within the TIME, offering a transformative perspective on tumor immunology and therapeutic responses (43). CD8+ T cells serve as critical effectors within the TIME that mediate antitumor immune responses during immunotherapy, whereas Tregs act as key regulators that suppress such responses. The limited efficacy of immunotherapy in PCa can be attributed to multiple factors, including the heterogeneity of PCa, its ‘cold’ tumor microenvironment, and the low number of neoantigens present (44). While CXCL12 enhances T cell migration to the TME and induces alterations in cell states, it concurrently recruits Tregs that suppress the cytotoxic functions of T cells (45). This phenomenon was corroborated in our study, where the spatial proximity between Tregs and CD8+ effector T cells was established, indicating that Tregs can perform direct immunosuppressive functions.

The CXCL12/CXCR4 axis is indeed a critical pathway for CD8+ T cell trafficking, but emerging evidence underscores the importance of complementary chemokine axes that contribute to immune cell recruitment and spatial coordination in the tumor microenvironment. Among these, the CXCR3 ligands (CXCL9/10/11) play a particularly pivotal role. CXCL9 and CXCL10 are induced by IFN-γ secreted from activated T cells and establish chemotactic gradients to recruit CXCR3+ CD8+ T cells to tumor niches. Dendritic cells, particularly DC1s, secrete CXCL9/10 and co-express CCL5, amplifying T cell infiltration and correlating with improved survival in solid tumors. For instance, CXCL9/10 secreted by DC1s establish chemotactic gradients that guide CXCR3+ CD8+ T cells to tumor niches, while their co-expression with CCL5 amplifies T cell infiltration and correlates with improved survival in solid tumors (46, 47). Another critical axis involves CXCL16 and its receptor CXCR6. CXCR6 is upregulated on effector T cells, enabling their localization to tumor stromal regions enriched in CCR7+ dendritic cells expressing CXCL16 (48). Similarly, the CCL5-CCR5 signaling pathway not only recruits CD8+ T cells directly but also activates an IFN-γ-driven positive feedback loop, sustaining CXCL9 production and enhancing antitumor immune responses (46). Targeting these chemokine axes in conjunction with CXCR4 inhibition presents a promising therapeutic strategy to synergistically optimize spatial immune remodeling within the TME and improve immunotherapy efficacy.

Regulatory T cells (Tregs), a CD4+ T cell subset with immunosuppressive functions, are characterized by the transcription factor FoxP3, high expression of CD25 (IL-2Rα), and CTLA-4 (49, 50). Tregs play a pivotal role in maintaining immune tolerance and preventing autoimmune diseases. However, within the TME, they are significantly enriched across various tumor types, contributing to immune evasion by suppressing antitumor immune responses through multiple mechanisms (51, 52). Tumor cells and stromal cells recruit Tregs to the tumor site primarily by secreting chemokines such as CCL22, CCL17 (binding to CCR4 on Tregs) (53), and CCL28 (binding to CCR10) (54). Once localized to the tumor microenvironment, Tregs secrete immunosuppressive cytokines, including TGF-β and IL-10, which directly impair the cytotoxic function of CD8+ effector T cells, thereby facilitating tumor immune evasion (55, 56). Furthermore, Tregs metabolically consume IL-2 within the TME through competitive uptake, leading to apoptosis of CD8+ effector T cells due to the absence of survival signals (57). Tregs are a major factor limiting the efficacy of immune checkpoint inhibitors, such as anti-PD-1 and anti-CTLA-4 therapies (51, 58). In the present study on PCa, a noncanonical chemotactic axis involving CCL22, CCL17, CCL28, Tregs, and CD8+ effector T cells was identified. Targeting this axis offers a dual therapeutic opportunity. First, inhibition of this axis can reduce Treg accumulation within the PCa TME. Second, it can minimize Tregs’ competitive consumption of IL-2, thereby increasing the local availability of IL-2 and restoring the cytotoxic function of CD8+ effector T cells. Consequently, this approach has potential to reprogram the immune landscape of PCa tumors, enhancing patient responsiveness to immunotherapy by simultaneously reducing Treg recruitment and reinvigorating antitumor immune activity.

Interleukin-2 therapy represents a promising strategy in cancer immunotherapy, with several IL-2 compounds developed to target the IL-2 receptor to enhance immunologic efficacy (59). IL-2 can specifically reinvigorate exhausted CD8+ T cells within the TME and augment their effector capabilities, demonstrating superior efficacy compared to PD-1 blockade, both quantitatively and qualitatively (60). Conversely, recent findings reveal that Tregs can emulously deplete IL-2, leading to dysfunction in intratumoral CD8+ T cells (27). Additionally, while CD8+ effector T cells lack sufficient stimulation from IL-2, Tregs exhibit a robust response within the PCa TME. This dynamic indicates that Tregs not only exert immunosuppressive functions autonomously but also compete for IL-2, inadvertently promoting tumor progression in PCa (61). Consequently, a new CD8-targeted IL-2 molecule has been developed that demonstrates over a 500-fold preference for CD8+ T cells compared to Tregs. This innovative compound effectively bypasses the limitations imposed by conventional IL-2 signaling on CD8+ T cells and significantly enhances antitumor activity (62). IL-2 activates signaling pathways via Janus kinases and the transcription factor STAT5, thereby regulating the differentiation and homeostasis of both pro-inflammatory and anti-inflammatory T cell subsets. Beyond its role in transcriptional programming, IL-2 serves as a critical regulator of T cell metabolism. Through STAT5 activation, IL-2 induces the expression of cytotoxic molecules in CD8+ T cells, including perforin, granzyme B, and IFN-γ, thereby enhancing their cytotoxic activity (63). STAT5 also binds directly to regulatory regions of the FoxP3 gene, promoting its transcription and ensuring the stability of FoxP3, which is essential for maintaining the immunosuppressive function of Tregs. In peripheral tissues, persistent IL-2-mediated activation of STAT5 supports the proliferation and survival of Tregs (64). Collectively, the IL-2/STAT5 axis plays an indispensable role in modulating lymphocyte fate and function, significantly influencing the tumor immune microenvironment (65). The functionality of tissue-resident CD8+ T cells is unaffected by this mechanism, though their exhausted state may arise from prolonged antigenic stimulation.

Our study combines computational and experimental approaches to uncover potential mechanisms of immune dysfunction within the TME of PCa. Specifically, we explored the role of the CXCL12/CXCR4 chemotactic axis in mediating Treg recruitment and its spatial colocalization with CD8+ effector T cells, alongside the contribution of IL-2 signaling to CD8+ T cell dysfunction via the STAT5/TOX pathway. Using integrated single-cell and spatial transcriptomics, we proposed that these interactions drive immune suppression in localized PCa. Furthermore, mechanistic insights were supported by *in vivo* and *in vitro* experiments presented in **Figure 8**, which provide preliminary evidence of the therapeutic potential of disrupting these pathways.

Our experimental findings indicated that CXCR4 inhibitor treatment reduced Treg infiltration into the TME and increased the abundance of functional CD8+ effector T cells. Additionally, IL-2 supplementation partially reversed the dysfunctional state of CD8+ effector T cells, as reflected by their cytotoxic activity in co-culture experiments. When these interventions were combined, tumor weight and volume were significantly reduced, supporting the hypothesis that simultaneous disruption of the CXCL12/CXCR4 axis and IL-2 modulation could reshape immune functionality within the TME. However, while these results are encouraging, they should be interpreted with caution and viewed as preliminary evidence rather than conclusive validation of the proposed mechanisms. Despite the use of both computational and experimental validations, several limitations remain. While our *in vivo* experiments confirmed the potential impact of CXCR4 inhibitor and IL-2 treatments on modulating immune cell dynamics and restricting tumor progression, the precise molecular mechanisms orchestrating these changes require further investigation. For example, the causal relationship between IL-2 signaling, STAT5 activation, and TOX upregulation in CD8+ effector T cells remains to be definitively established. Future studies involving direct measurements of pSTAT5 activation, genetic perturbation experiments targeting STAT5, and depletion assays for IL-2 within the TME would strengthen the proposed mechanistic framework. Additionally, it will be critical to validate these findings in a larger cohort of *in vivo* models and in patient-derived samples to enhance translational relevance.

In conclusion, our study provides a multi-dimensional analysis of the immune landscape in localized PCa, integrating computational predictions with wet-lab experiments to propose critical interactions within the TME that promote immune evasion. While our results highlight the therapeutic potential of targeting the CXCL12/CXCR4 axis and IL-2 signaling, further experimental validation is necessary to establish causality and evaluate the feasibility of translating these findings into clinical applications. We believe these insights lay the groundwork for future studies aimed at developing innovative therapeutic strategies for improving outcomes in PCa patients.

## Data Availability

The original contributions presented in the study are included in the article/**Supplementary Material**. Further inquiries can be directed to the corresponding author.
