# Supplementary figures and images for: CXCL12/CXCR4 axis governs Treg spatial dominance over CD8+ T cells via IL-2 sequestration: a dual therapeutic target in prostate cancer
Source: Front Immunol. 2025 Jul 8;16:1626708. doi: 10.3389/fimmu.2025.1626708 (PMC12279891; doi:10.3389/fimmu.2025.1626708)

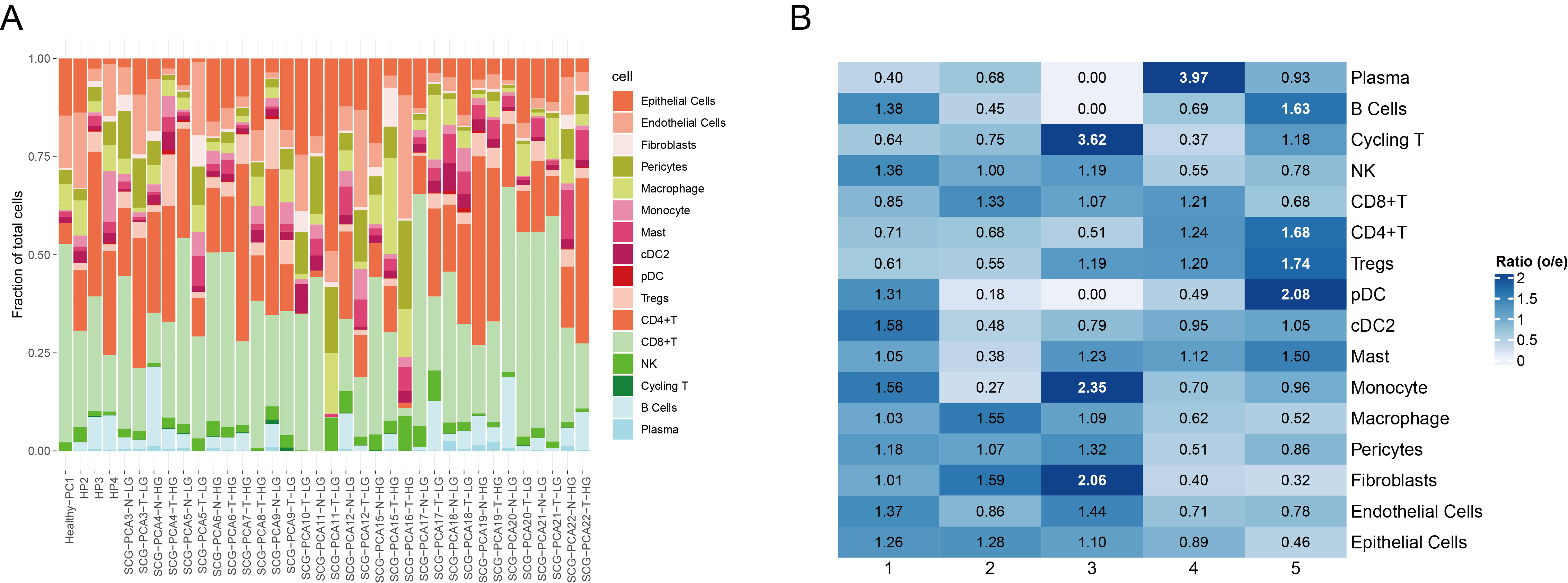

Supplement: Supplementary file 2 [file Image1.jpeg]

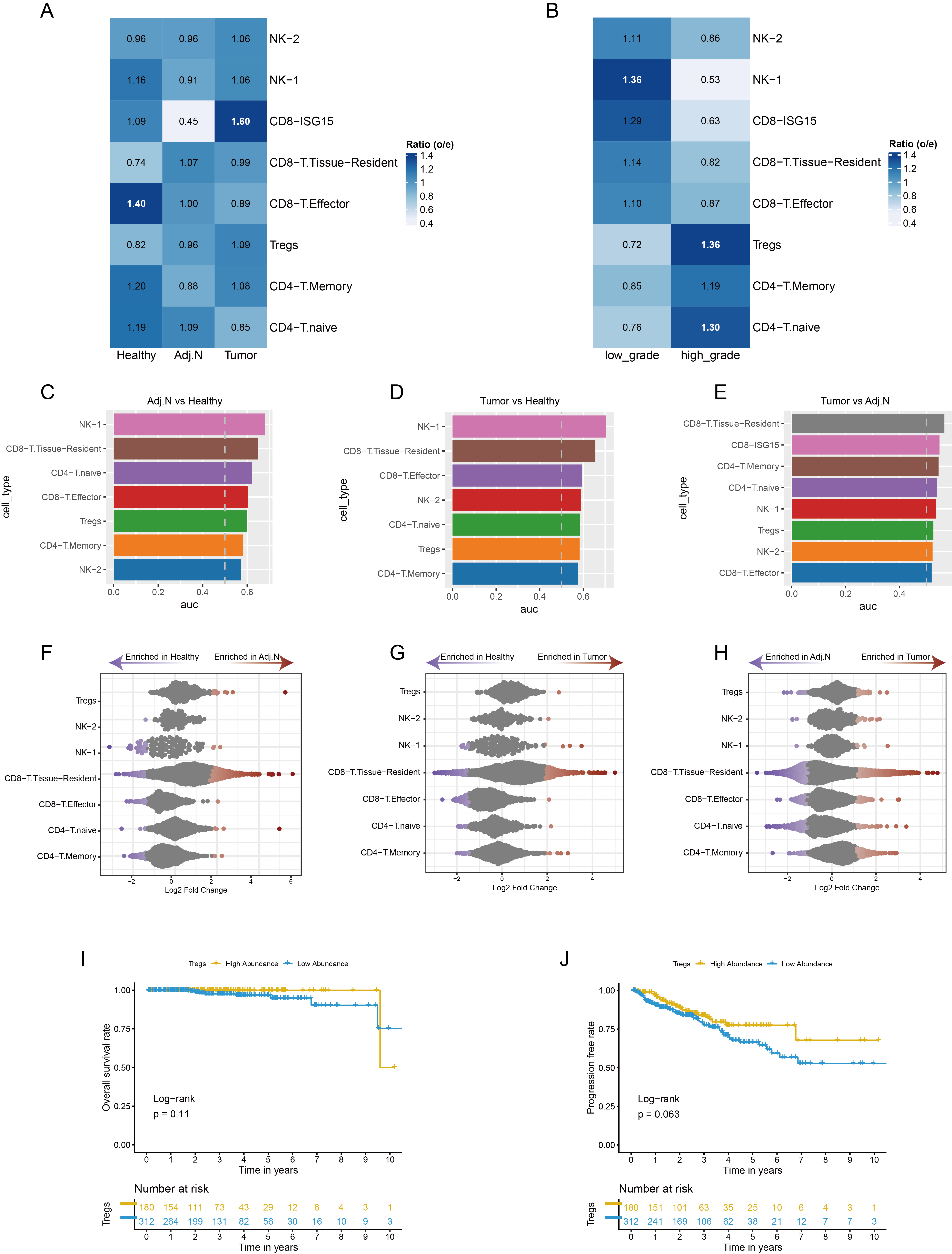

Supplement: Supplementary file 3 [file Image2.jpeg]

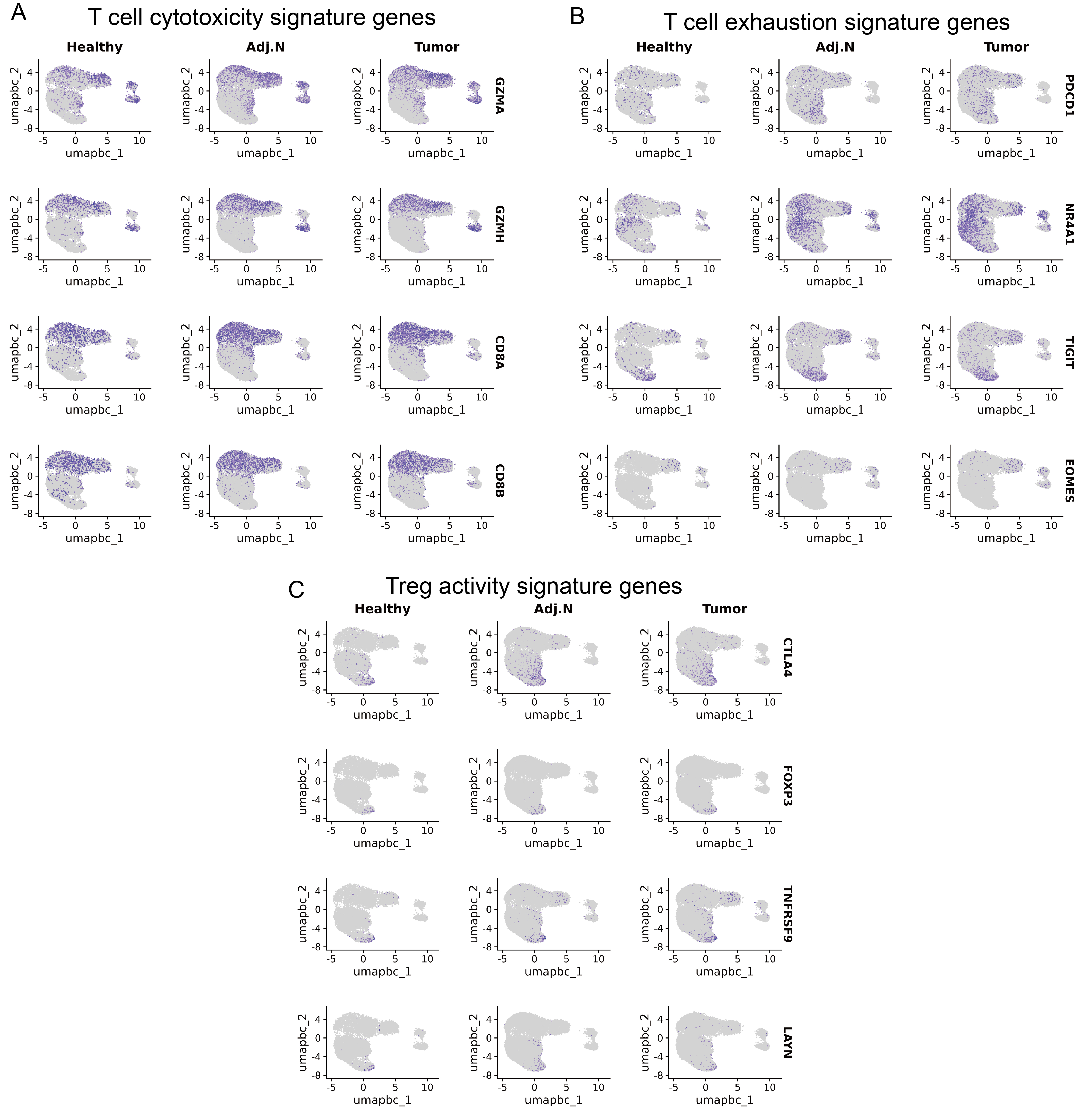

Supplement: Supplementary file 4 [file Image3.jpeg]

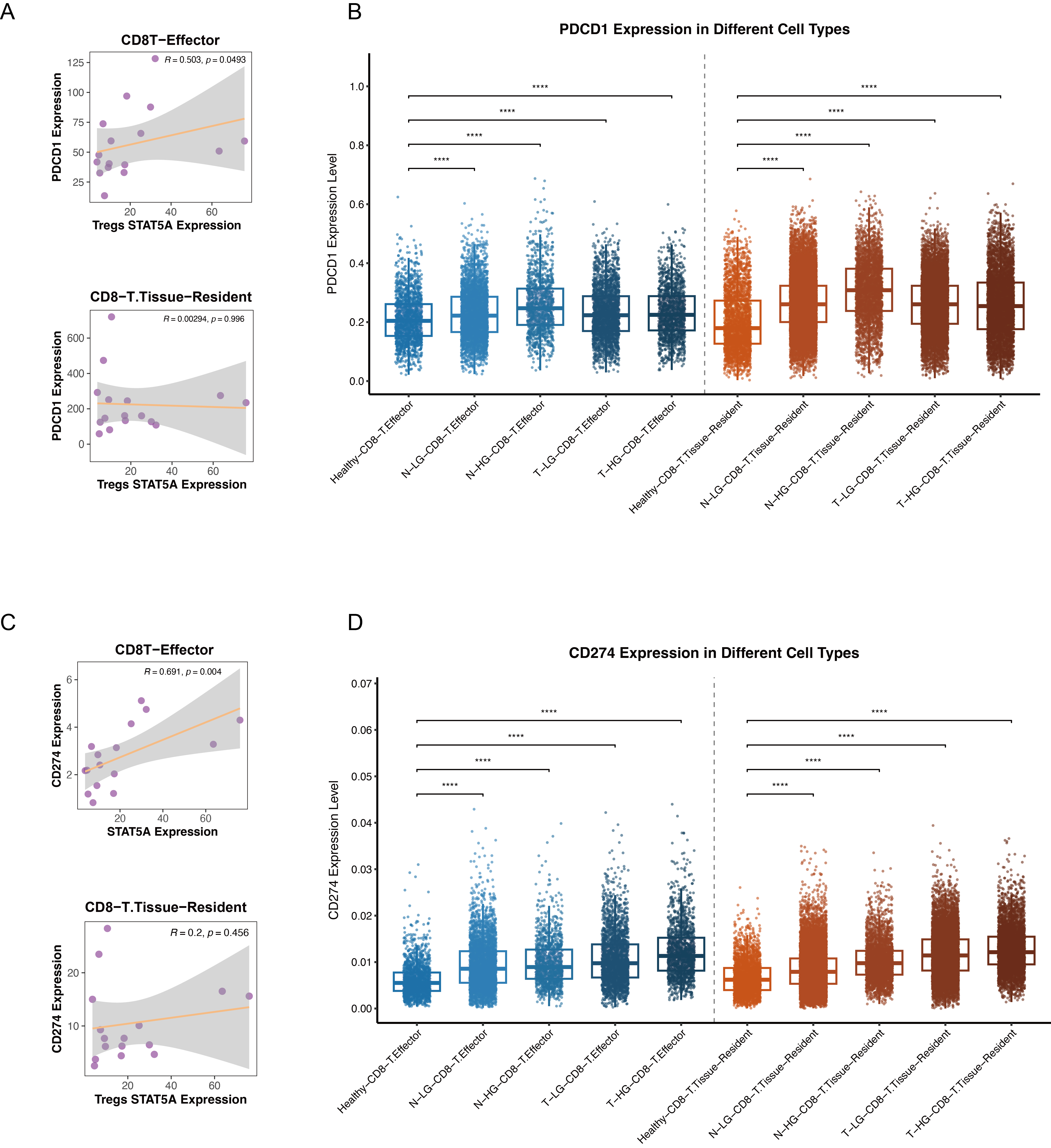

Supplement: Supplementary file 5 [file Image4.jpeg]
